# Screening for bilayer-active and likely cytotoxic molecules reveals bilayer-mediated regulation of cell function

**DOI:** 10.1085/jgp.202213247

**Published:** 2023-02-10

**Authors:** Thasin A. Peyear, Olaf S. Andersen

**Affiliations:** 1 Department of Physiology and Biophysics, Weill Cornell Medicine, New York, NY, USA; 2 Graduate Program in Physiology, Biophysics and Systems Biology, Weill Cornell Graduate School of Medical Sciences. New York, NY, USA

## Abstract

A perennial problem encountered when using small molecules (drugs) to manipulate cell or protein function is to assess whether observed changes in function result from specific interactions with a desired target or from less specific off-target mechanisms. This is important in laboratory research as well as in drug development, where the goal is to identify molecules that are unlikely to be successful therapeutics early in the process, thereby avoiding costly mistakes. We pursued this challenge from the perspective that many bioactive molecules (drugs) are amphiphiles that alter lipid bilayer elastic properties, which may cause indiscriminate changes in membrane protein (and cell) function and, in turn, cytotoxicity. Such drug-induced changes in bilayer properties can be quantified as changes in the monomer↔dimer equilibrium for bilayer-spanning gramicidin channels. Using this approach, we tested whether molecules in the Pathogen Box (a library of 400 drugs and drug-like molecules with confirmed activity against tropical diseases released by Medicines for Malaria Venture to encourage the development of therapies for neglected tropical diseases) are bilayer modifiers. 32% of the molecules in the Pathogen Box were bilayer modifiers, defined as molecules that at 10 µM shifted the monomer↔dimer equilibrium toward the conducting dimers by at least 50%. Correlation analysis of the molecules’ reported HepG2 cell cytotoxicity to bilayer-modifying potency, quantified as the shift in the gramicidin monomer↔dimer equilibrium, revealed that molecules producing <25% change in the equilibrium had significantly lower probability of being cytotoxic than molecules producing >50% change. Neither cytotoxicity nor bilayer-modifying potency (quantified as the shift in the gramicidin monomer↔dimer equilibrium) was well predicted by conventional physico-chemical descriptors (hydrophobicity, polar surface area, etc.). We conclude that drug-induced changes in lipid bilayer properties are robust predictors of the likelihood of membrane-mediated off-target effects, including cytotoxicity.

## Teaser

Drug-induced changes in bilayer properties alter the function of diverse membrane proteins, thereby changing cell function, which may cause cytotoxicity. Drug-induced changes in bilayer properties can be quantified as shifts in the gramicidin monomer↔dimer equilibrium, which allows for quantifying the likelihood a drug is cytotoxic.

## Introduction

Many biologically active (bioactive) molecules, including drugs, are amphiphiles that partition into the lipid bilayer component of cellular membranes thereby altering bilayer physical properties like elasticity, curvature, and thickness (Seddon, 1990; Evans et al., 1995; Zhelev, 1998; Chen et al., 2003; Lundbaek et al., 2005; Marsh, 2008), which will alter the bilayer contribution to the energetic cost of membrane protein conformational changes that involve the proteins’ bilayer-spanning domains and, in turn, membrane protein function (Lundbaek et al., 2005; Lundbaek et al., 2010a; Rusinova et al., 2011; Ingólfsson et al., 2014). The functional consequences of such changes in bilayer properties (changes in the bilayer contribution to the energetics of a conformational change) can be quantified in studies on well-defined reporter proteins (Gruner, 1991; Brown, 1994; Lundbaek et al., 2010a; Ingólfsson et al., 2014), which show that an amphiphile may alter the activity of functionally and structurally diverse membrane proteins at similar concentrations (Ingólfsson et al., 2014). Thus, if one membrane protein is modulated by a bioactive amphiphile (at some concentration), then many other membrane proteins will be modulated at similar concentrations. These indiscriminate changes in membrane protein function are likely to compromise cellular homeostasis and, if the changes in function are of sufficient magnitude, cause cytotoxicity.

We explored this question in the context of drug development against neglected tropical diseases (NTDs), a group of communicable diseases that are prevalent in tropical and subtropical countries (Fürst et al., 2017; World Health Organization, 2017). To promote the development of effective/inexpensive treatments for the most common NTDs, the Medicines for Malaria Venture (MMV) compiled and released the Pathogen Box, an open source project containing 400 drugs and drug-like molecules (drugs for short) with confirmed activity against NTDs, to catalyze a collaborative environment for drug discovery and development (MMV, 2014), which led to the identification of numerous leads for treating NTDs, e.g., Veale, 2019.

All drugs in the Box have been characterized in terms of their biological activity and deemed to be suitable for an initial drug discovery program. MMV also provides extensive cheminformatics plus information about biological activity, including cytotoxicity, on the drugs in the Box (MMV, 2017). Among the selection criteria for including drugs in the Pathogen Box was whether they were deemed to have appropriate physicochemical properties (Hughes et al., 2008; Price et al., 2009; Waring et al., 2015), including the calculated octanol/water partition coefficient and polar surface area.

Understanding how a molecule’s physicochemical properties may relate to cytotoxicity is likely to improve drug design and development (Leeson and Springthorpe, 2007; Leeson, 2012); yet, predicting the likelihood that a drug candidate may be (cyto)toxic based on its physicochemical characteristics remains a challenge (Waring et al., 2015). It is in this context important that many drugs are amphiphiles that partition into the lipid bilayer component of cellular membranes, where they alter bilayer physical properties and thereby membrane protein function (Lundbaek et al., 1996; Lundbaek et al., 2004; Lundbaek et al., 2005; Artigas et al., 2006; Rusinova et al., 2011; Ingólfsson et al., 2014).

**Figure 1.**
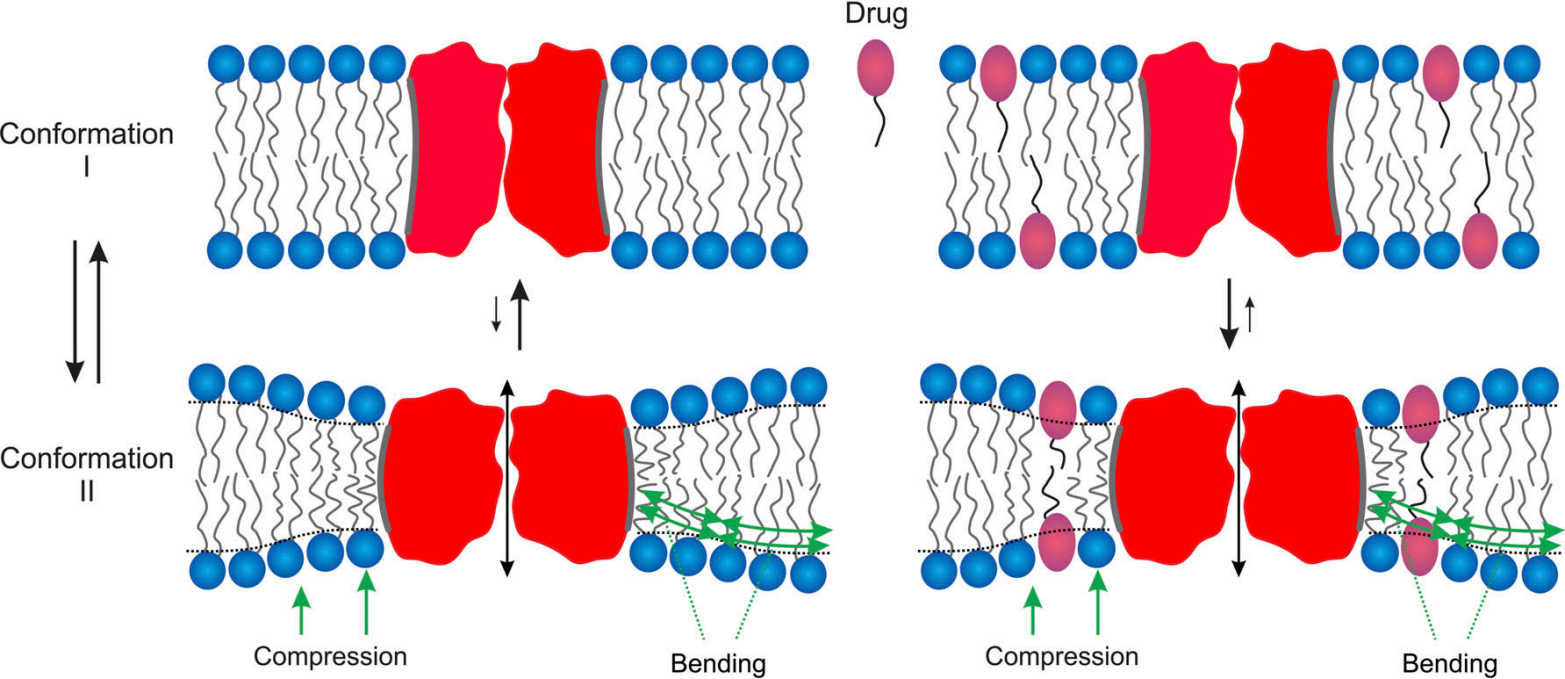
**Membrane proteins undergo conformational changes as part of their functional cycle.** When these changes involve the proteins bilayer-spanning domains, they will alter the packing of the adjacent lipids. These changes can to a first approximation can be describes as a local compression of each leaflet and bending of the bilayer/solution interface (indicated by the green arrows), which represent the major contributions to the energetic cost of the bilayer deformation, the bilayer deformation energy (Δ*G*_*def*_). Δ*G*_*def*_ varies with changes in the bilayer’s mechanical properties (elasticity, thickness, and intrinsic curvature), which change when an amphiphile/drug partitions into the bilayer/solution interface. The resulting change in Δ*G*_*def*_ will alter the equilibrium distribution between protein conformations I and II—and protein function.

This bilayer-mediated regulation of membrane protein function arises because the hydrophobic adaptation between membrane proteins and their host bilayer causes membrane proteins to be energetically coupled to their host bilayer (Gruner, 1991). Conformational changes (from, say, state I to state II) that involve the proteins’ bilayer-spanning domains (Fig. 1), therefore, will alter the organization of the adjacent lipids, which has an associated energetic cost (Huang, 1986; Gruner, 1991; Lundbaek et al., 2010a; Rusinova et al., 2011): $\Delta G_{\text{bilayer}}^{I\to \text{II}}=\Delta G_{def}^{\text{II}}-\Delta G_{def}^{I},$ where $\Delta G_{def}^{I}$ and $\Delta G_{def}^{II}$ denote the energetic cost of the local, protein-induced bilayer deformations associated with each state. Norimatsu et al. (2017) and Wang and Boudker (2020) show the existence of conformational changes in integral membrane proteins and their impact on the adjacent bilayer; Zhou et al. (2019) estimate the associated changes in $\Delta G_{\text{bilayer}}^{I\to \text{II}},$ which may be 6–7 kcal/mole. The total energetic cost of a conformational change from state I to state II $\left(\Delta G_{total}^{I\to \mathrm{II}}\right)$ thus will be the sum of contributions due to structural rearrangements within the protein $\left(\Delta G_{\mathrm{protein}}^{I\to \mathrm{II}}\right)$ and rearrangements within the bilayer: $\Delta G_{\mathrm{total}}^{I\to \mathrm{II}}=\Delta G_{\mathrm{protein}}^{I\to \mathrm{II}}+\Delta G_{\mathrm{bilayer}}^{I\to \mathrm{II}}$ plus other contributions (Rusinova et al., 2021). See also Section 1 in the supplemental text at the end of the PDF.

The functional consequences of changes in bilayer properties (changes in $\Delta G_{\mathrm{bilayer}}^{I\to \mathrm{II}})$ can be quantified using well-defined reporter proteins (Gruner, 1991; Brown, 1994; Lundbaek et al., 2010a; Ingólfsson et al., 2014). One such reporter is the gramicidin channel, which forms by transmembrane dimerization of two non-conducting subunits (Bamberg and Läuger, 1973; Zingsheim and Neher, 1974; O’Connell et al., 1990). Gramicidin channels are known to be modulated by changes in lipid bilayer properties, whether induced by changes in lipid composition (Kolb and Bamberg, 1977; Elliott et al., 1983) or by bioactive molecules (Haydon et al., 1977; Pope et al., 1982; Haydon and Urban, 1983; Elliott et al., 1985; Hwang et al., 2003; Lundbaek et al., 2004; Lundbaek et al., 2005; Artigas et al., 2006; Bruno et al., 2007; Ingólfsson and Andersen, 2010; Rusinova et al., 2011; Herold et al., 2014; Rusinova et al., 2015).

**Figure S1.**
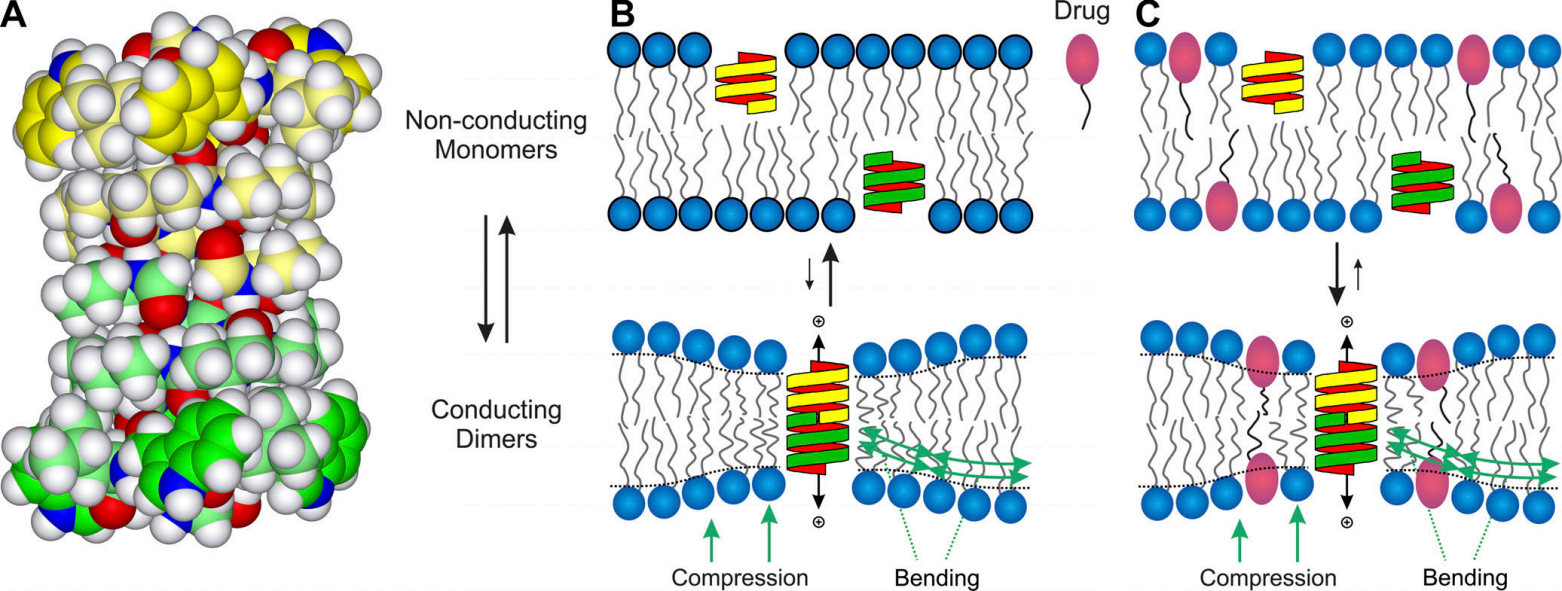
**Gramicidin channel structure and formation. (A)** Space-filling model of the β^6.3^-helical gramicidin A channel dimer (energy-minimized structure coordinates based on PDB accession nos. 1GRM [Arseniev et al, 1986b; Lomize et al, 1992], 1MAG [Ketcham et al, 1997], and 1JNO [Townsley et al, 2001]). The carbon atoms in the two subunits are green and yellow, respectively. **(B)** Gramicidin channels form by transmembrane dimerization of two non-conducting subunits (O’Connell et al., 1990). **(C)** When an amphiphile/drug partitions into the bilayer/solution interface it will alter bilayer physical properties (elasticity, thickness, and intrinsic curvature) and thereby change the bilayer deformation energy associated with channel formation, which in turn will shift the gramicidin monomer↔dimer equilibrium, usually toward the right.

Changes in bilayer properties (elasticity, thickness, and intrinsic curvature) will alter the gramicidin monomer↔dimer equilibrium (Fig. S1), which can be measured as changes in the number of conducting channels per unit membrane area: changes in appearance rates and lifetimes of bilayer-spanning gramicidin channels (Sawyer et al., 1989; Lundbaek et al., 2010a); or changes in the time course of fluorescence quenching in fluorophore-loaded large unilamellar phospholipid vesicles (LUVs) that have been doped with gramicidin and mixed with a gramicidin channel-permeant quencher, Tl^+^ (Ingólfsson and Andersen, 2010); see also Section 1 at the end of the PDF. Bilayer-perturbing effects can be generalized to other lipid compositions (Bruno et al., 2007; Rusinova et al., 2011; Rusinova et al., 2015; Herold et al., 2017), cells (Lin and Will, 2012), and systems (Hughes et al., 2008). Changes in bilayer properties, as evaluated using gramicidin channels, can also be used to predict changes in function of other membrane proteins (Lundbaek et al., 2005; Rusinova et al., 2011; Ingólfsson et al., 2014; Herold et al., 2017) demonstrating the generality of this mechanism.

We show that drug-induced changes in fluorescence quench rate correlate with the drugs’ likely cytotoxicity, reported by MMV (2017) as the concentration that produces 20% inhibition of HepG2 cell growth (HepG2 *CC*_20_). Drugs with higher quench rates tend to have lower *CC*_20_ values, meaning they are more likely to be cytotoxic. Drugs may, of course, be cytotoxic for reasons not associated with the membrane, but bilayer-modifying potency is a promising predictor of off-target effects and cytotoxicity. Although physico-chemical properties allow for predicting drug-likeliness (e.g., Bickerton et al., 2012), we found they are less effective in predicting off-target effects and cytotoxicity. Our results, taken together with earlier studies demonstrating that that drug-induced changes in ion channel function correlate with changes in gramicidin channel function (summarized in the previous paragraph), suggest that testing for bilayer-modifying potency will help identify membrane-mediated off-target effects when using amphiphiles (including drugs) to manipulate membrane protein or cell function.

## Materials and methods

### Materials

1,2–Dierucoyl–*sn*–glycero–3–phosphocholine (DC_22:1_PC) in chloroform (25 mg/ml) was >99% pure from Avanti Polar Lipids. Methanol ≥99.8% was from VWR. Thallium nitrate (TlNO_3_) ≥99.9%, sodium nitrate (NaNO_3_) ≥99%, HEPES ≥99.5%, bovine serum albumin (BSA) ≥98%, 3-[(3-cholamidopropyl)-dimethylammonium]-1-propanesulfonate (CHAPS) ≥98%, and gramicidin from *Bacillus aneurinolyticus* (*Bacillus brevis*) ≥95% were from Sigma–Aldrich Co. The di-sodium salt of 8–aminonaphthalene–1,3,6–trisulfonate (ANTS) was from Invitrogen. The Pathogen Box was a gift from the Medicines for Malaria Venture: https://www.mmv.org/mmv–open/pathogen–box. The drugs were provided as 10 µl aliquots of 10 mM drug dissolved in DMSO and used as supplied.

Stock solutions of buffers and quenchers were prepared ahead of the experiment and, unless otherwise noted, were dissolved in deionized water, and adjusted to pH 7 with sodium hydroxide (NaOH) and nitric acid (HNO_3_). Na–ANTS buffer was 25 mM ANTS, 100 mM NaNO_3_, and 10 mM HEPES; it was stored shielded from light. Na buffer was 140 mM NaOH and 10 mM HEPES. Tl quench buffer was 50 mM TlNO_3_, 94 mM NaNO_3_, and 10 mM HEPES. All buffer and quencher stock solutions were stored at 12.5°C; the DC_22:1_PC was stored at −40°C.

### Methods

#### Gramicidin channels

The naturally occurring mixture of the linear gramicidins from *Bacillus brevis* has historically been called gramicidin D (gD), after R. Dubos, who discovered the gramicidins (Dubos, 1939); it contains 80–85% [Val^1^] gramicidin A (gA), 6–7% gramicidin B (gB), [Val^1^, Phe^11^]gA, and 5–14% gramicidin C (gC), [Val^1^, Tyr^11^]gA (Abo-Riziq et al., 2006). We used the gD as a 500 μg/ml (265 µM) solution in methanol, which was stored at −40°C.

Gramicidin channels are formed by transmembrane dimerization of two non-conducting gramicidin subunits (O’Connell et al., 1990; Lum et al., 2017; Fig. S1). gA, gB, and gC form structurally equivalent anti-parallel, dimeric channels with very similar properties (Sawyer et al., 1990), meaning that approximately two-thirds of the measured ion flux will be through symmetric gA/gA homodimeric channels, approximately one-fifth will be through asymmetric gA/gB, or gA/gC heterodimeric channels; the remaining will be through symmetric gB/gB and gC/gC homodimeric channels and asymmetric gB/gC heterodimeric channels. Experiments using the readily available gD give similar results as experiments with purified gA (Sun et al., 2020). Because the channels’ hydrophobic length is less than the host bilayer’s hydrophobic thickness, channel formation produces a local bilayer thinning (Fig. S1), which incurs an energetic cost (Huang, 1986; Lundbaek et al, 2010a).

Gramicidin was incorporated into LUVs that encapsulate the aqueous fluorophore ANTS (Ingólfsson and Andersen, 2010), which is quenched by the gramicidin channel-permeant heavy monovalent cation thallium (Tl^+^). When a drug is added and allowed to equilibrate with the LUVs, the drug will partition into the vesicle bilayer, which will alter bilayer properties, usually decreasing the bilayer stiffness (ease of deformation). Such drug-induced bilayer softening will decrease the energetic cost of dimerization and shift the monomer↔dimer equilibrium toward the conducting dimers, and drug-induced stiffening of the bilayer will increase the energetic cost of dimerization and shift the monomer↔dimer equilibrium toward the non-conducting monomers. These shifts in the monomer↔dimer equilibrium can be evaluated using stopped-flow spectrofluorometry (see below).

#### LUVs

ANTS-loaded LUVs incorporating gD (Ingólfsson and Andersen, 2010) were prepared using gD and DC_22:1_PC (molar ratio 1:2,000), which were mixed in a 50-ml round-bottom flask, dried to a thin film under nitrogen to remove the chloroform and methanol, then further dried under a vacuum overnight to remove any remaining solvent. The lipid film was rehydrated in Na–ANTS buffer to give a 10 mM lipid suspension, which was thoroughly vortexed (the round-bottom flask was covered and protected from light for the duration of the LUV preparation and experiment). The suspension was incubated at room temperature for at least 3 h, followed by sonication for 1 min at low power. The resulting suspension then was subjected to six freeze–thaw cycles using dry ice (10 min) and 45–55°C water (5 min); after each cycle, the sample was thoroughly vortexed. At the end of the sixth cycle, the resulting multilamellar vesicle (MLV) suspension was extruded 20 times (passes through the filter) at room temperature through a 10 ml LIPEX Extruder (Northern Lipids Inc) with a 0.1 μm polycarbonate filter and a 25-mm polyester drain disc. The resulting LUV suspensions were stored at −40°C and could be used for at least 1 mo. Before use, extravesicular ANTS was removed using a PD–10 desalting column (GE Healthcare); these LUVs (5 mM lipid suspension) were stored at 12.5°C and used within 7 d.

#### Dynamic light scattering

**Figure S2.**
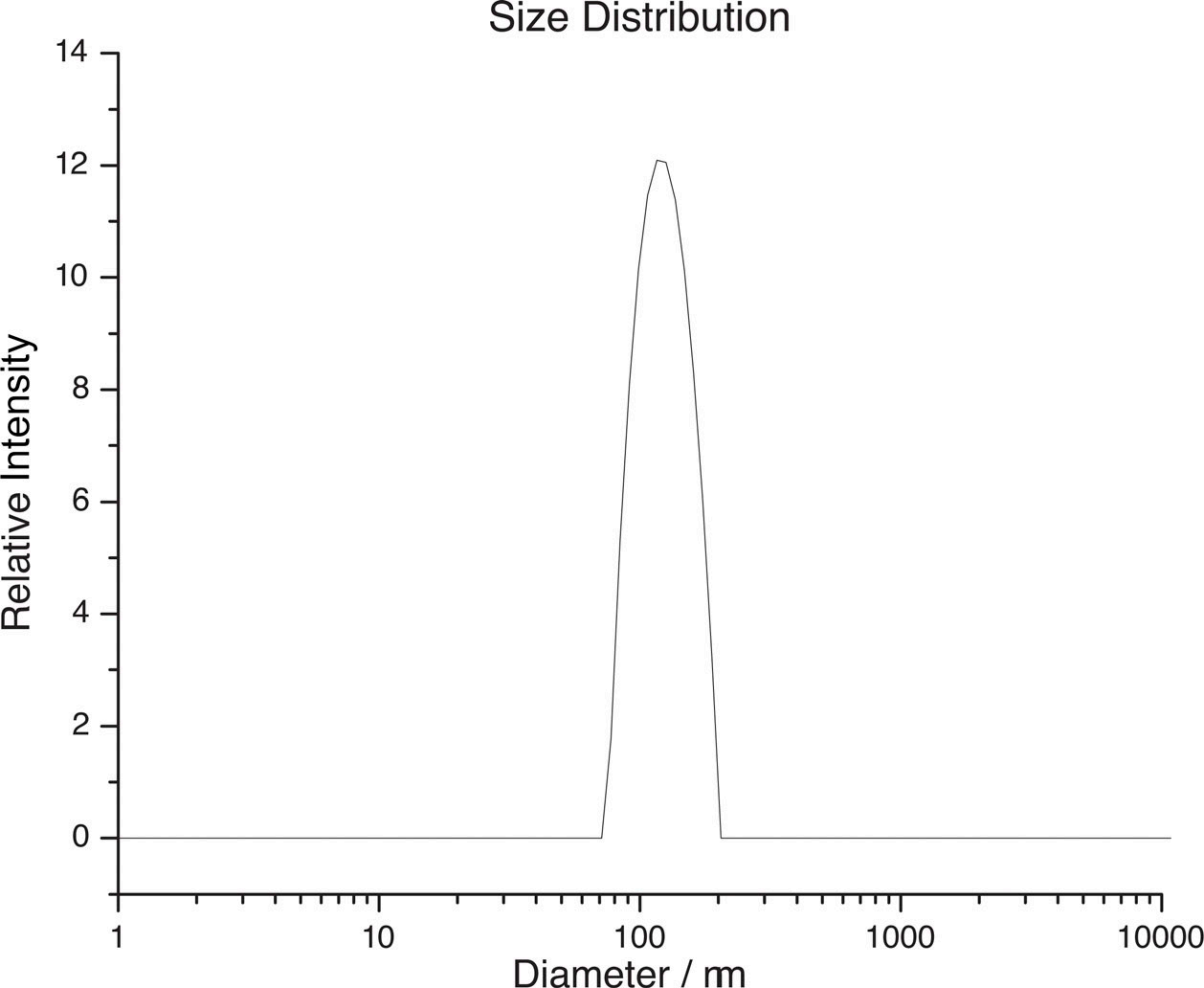
**The distribution of LUV diameters for vesicles used in the fluorescence quench experiments; results for one LUV preparation.** The average hydrodynamic diameter was 130 ± 5 nm and the polydispersity index (PDI) was 0.09 ± 0.04 (*n* = 9).

The LUV size distribution was determined using a Litesizer 500 dynamic light scattering instrument with the Kalliope software (Anton Paar). Using disposable cuvettes with 1.0 ml sample volume and a lipid concentration of 50 μM in Na buffer, the transmittance was ≈88%. The refractive index and viscosity of the Na buffer were set to 1.3318 and 0.9064 cP, respectively, the default settings in Kalliope. The default correlation function and fitting curve were used to calculate the diffusion coefficient, mean hydrodynamic diameter (*d*_LUV_), and the polydispersity index (PDI), defined as ${\left(\sigma /d_{\text{LUV}}\right)}^{2}\text{,}$ where σ^2^ denotes the variance of the size distribution (e.g., Clayton et al., 2016). Each sample was tested 1 d after extrusion with three independent measurements and at least seven repeats in each measurement. There was only one discernable peak (Fig. S2), with *d*_LUV_ = 130 ± 5 nm and a PDI of 0.09 ± 0.04 (*n* = 9). A PDI < 0.1 is considered to indicate a monodisperse sample (Clayton et al., 2016). For an LUV sample with *d*_LUV_ = 130 nm and a PDI = 0.06, 10% of the LUVs will have a diameter <87 nm and 10% of the LUVs will have a diameter >160 nm.

#### Physicochemical parameters and biological activity

Most of the physicochemical molecule properties used in the analysis of the results were estimated using the Schrödinger Suite (Schrödinger). Polar surface area (PSA), defined as the Van der Waals surface area of polar nitrogen and oxygen atoms, was calculated using QikProp. Hydrophobicity was estimated using ALogP, which was calculated using fragmental methods (Ghose et al., 1998). ALogP and the aromatic ring count were evaluated using Canvas. Data for the in vitro fraction unbound in mouse plasma (*fu*_mouse_), or human microsomal protein (*fu*_mic_), were from (MMV, 2017). Toxicity information was provided by MMV, as the drug concentration that causes death of 20% of cells (*CC*_20_) in the human liver cancer cell line HepG2.

#### Stopped-flow spectrofluorometry

The time course of ANTS fluorescence quench was measured at 25°C using an SX–20 stopped-flow spectrofluorometer (Applied Photophysics) in the single mixing mode. The excitation wavelength was 352 nm and the emission above 450 nm was recorded using a high-pass filter and a sampling rate of 5,000 points/s. The measured instrument deadtime was ∼1.2 ms. Samples were prepared by diluting the ANTS-LUV stock suspension 100-fold with Na buffer to 50 μM lipid; for each sample, an aliquot of the drug in question was added to a final concentration of 10 μM and allowed to equilibrate for 10 min before testing (the final DMSO concentration in the mixture was 12.8 mM, or 0.1%, a concentration at which DMSO has no effect on bilayer properties; Ingólfsson and Andersen, 2010). For each sample, 8 1-s control mixing reactions were recorded by mixing the LUV suspension with Na buffer (no Tl^+^), followed by 10 1-s mixing reactions with the Tl quench buffer. “Bad” traces, which may occur for reasons such as air bubbles, were removed based on visual inspection. Each drug was evaluated using two independently prepared LUV preparations; the quality of each batch was evaluated using the control rate, in the presence of 0.1% DMSO with no added drug (negative control), and the increase in quench rate observed with 5% ethanol (positive control).

To evaluate the possible effects of protein binding, some drugs were retested in the presence of an additional 60 µM BSA (corresponding to the BSA concentration in cell culture media supplemented with 10% fetal calf serum).

#### Data analysis

The rate of Tl^+^ influx was quantified from the time course of fluorescence quench using the Stern–Volmer relationship for dynamic quenching (Moore and Raftery, 1980; Ingólfsson and Andersen, 2010). Due to the unavoidable variations in LUV size (surface area and volume) and surface density of conducting channels in the LUV membranes, the volume-averaged change in [Tl^+^]_i_ cannot be described by a single exponential function. [Tl^+^]_i_ will increase faster in the smaller LUVs and slower in the larger LUVs (Ingólfsson and Andersen, 2010), and the increase in the volume-averaged [Tl^+^]_i_ will be a weighted sum of exponential functions*,* which can be expressed in a mathematically convenient form by a so-called modified stretched exponential function (Berberan-Santos et al., 2005); for details, see Section 2 at the end of the PDF.

The time course of fluorescence quench can be expressed as (Eq. S17):

$$\frac{F\left(t\right)}{F\left(0\right)}=\frac{1}{1+K_{\text{SV}}\cdot {\left[{\text{Tl}}^{+}\right]}_{i}\left(t\right)}=\frac{1}{1+K_{\text{SV}}\cdot {\left[{\text{Tl}}^{+}\right]}_{e}\cdot \left(1-\exp\left\{1-{\left(1+t/\tau_{0}\right)}^{\beta }\right\}\right)}\text{,}$$ (1)

where *F*(*t*) denotes the fluorescence intensity as function of time, *t*; *F*(0) is the fluorescence intensity at time 0, before any quench has occurred; *K*_SV_ is the Stern–Volmer coefficient (60 M^–1^ for Tl^+^; Ingólfsson and Andersen, 2010); [Tl^+^]_i_(t) is the intravesicular Tl^+^ concentration (as function of time); and [Tl^+^]_e_ is the extravesicular Tl^+^ concentration. τ_0_ (τ_0_ > 0) is a parameter with units of time and β (0 < β ≤ 1) is a parameter reflecting the dispersity of vesicle volumes, areas, and surface density of conducting gramicidin channels (β = 1 for a homogenous population of LUVs). The initial quench rate at *t* = 0, *Rate*(0) is given by (cf. Eq. S18):

$$\begin{array}{l} Rate\left(0\right)={\frac{d}{dt}\frac{F\left(t\right)}{F\left(0\right)}|}_{0}=-K_{\text{SV}}\cdot {\left[{\text{Tl}}^{+}\right]}_{e}\cdot \frac{\beta }{\tau_{0}}\cdot \\ \frac{exp\left\{1-{\left(1+t/\tau_{0}\right)}^{\beta }\right\}}{{\left\{1+K_{\text{SV}}\cdot {\left[{\text{Tl}}^{+}\right]}_{e}\cdot \left(1-exp\left\{1-{\left(1+t/\tau_{0}\right)}^{\beta }\right\}\right)\right\}}^{2}}|=-K_{\text{SV}}\cdot {\left[{\text{Tl}}^{+}\right]}_{e}\cdot \frac{\beta }{\tau_{0}}\text{,} \end{array}\;$$ (2)

where ${\left[{\text{Tl}}^{+}\right]}_{e}\cdot \beta /\tau_{0}$ is the initial rate of Tl^+^ influx (cf. Eqs. S10 and S19).

In practice, there are three separate ANTS-containing (and fluorescent) compartments: first, the ANTS in the extravesicular solution, which will be quenched rapidly, within the time resolution of the instrument; second, LUVs with conducting channels that can be quenched; third, vesicles that cannot be quenched because they either are LUVs without any conducting gramicidin channels (during the 1 s measurement), or multilamellar vesicles (e.g., Scott et al., 2019). Tl^+^ can cross the LUV membrane, as TlNO_3_ ion pairs (Martinus and Vincent, 1976) and, maybe, through transient membrane defects (Paula et al., 1996), which gives rise to slow quench of the ANTS fluorescence (Ingólfsson and Andersen, 2010); this has no significance for quench rates measured over the first 1 s of mixing and is not considered further.

The fluorescence signal recorded when mixing the LUVs with Na buffer, *F*(*t* = 0,b), where nothing is quenched, is the sum of the initial fluorescence from the three compartments:

F(t=0,b)=F(extravesicular)+F(intravesicular)+F(unquenchable). (3)

Only the second group of LUVs with conducting channels is of interest; the other two groups contribute to the signal, however, and it becomes convenient to explicitly consider the three compartments and their contributions to the fluorescence signal: *F*(extravesicular), the fluorescence signal from the extravesicular ANTS; *F*(intravesicular), the fluorescence signal from the quenchable LUVs; and *F*(unquenchable), the fluorescence signal from the population of unquenched vesicles. These three contributions can be quantified as follows:

First, the fluorescence signal recorded immediately after mixing the LUV suspension with Tl quench buffer, *F*(*t* = 0,q), will be less than *F*(*t* = 0,b) because the extravesicular ANTS will be quenched “instantly,” within the dead time of the instrument:

$$F\left(\text{extravesicular}\right)=\left[F\left(0,b\right)-F\left(0,q\right)\right]\frac{1+K_{\text{SV}}\cdot {\left[{\text{Tl}}^{+}\right]}_{e}}{K_{\text{SV}}\cdot {\left[{\text{Tl}}^{+}\right]}_{e}}\text{.}$$ (4)

Second, the fluorescence recorded “long” after mixing the LUV suspension with the Tl^+^ quencher, *F*(*t* = ∞,q) will be the sum of the quenched signal from the extravesicular ANTS and the population of quenchable vesicles, plus the signal from the unquenched vesicles (and any other non-quenching elements):

$$F\left(t=\infty ,q\right)=\frac{F\left(\text{extravesicular}\right)+F\left(\text{intravesicular}\right)}{1+K_{\text{SV}}\cdot {\left[{\text{Tl}}^{+}\right]}_{e}}+F\left(\text{unquenchable}\right),$$ (5)

because [Tl^+^]_i_(∞) in the quenchable vesicles will be equal to [Tl^+^]_e_.

Combining Eqs. 3, 4, and 5, *F*(extravesicular), *F*(intravesicular), and *F*(unquenchable) can be expressed in terms of the experimental observables, *F*(t = 0,b), *F*(t = 0,q), and *F*(t = ∞,q):

$$F\left(\text{extravesicular}\right)=\left[F\left(0,b\right)-F\left(0,q\right)\right]\cdot \frac{1+K_{\text{SV}}\cdot {\left[{\text{Tl}}^{+}\right]}_{e}}{K_{\text{SV}}\cdot {\left[{\text{Tl}}^{+}\right]}_{e}}\text{,}$$ (6)

$$F\left(\text{intravesicular}\right)=\left[F\left(0,q\right)-F\left(\infty ,q\right)\right]\cdot \frac{1+K_{\text{SV}}\cdot {\left[{\text{Tl}}^{+}\right]}_{e}}{K_{\text{SV}}\cdot {\left[{\text{Tl}}^{+}\right]}_{e}}\text{,}$$ (7)

and

$$F\left(\text{unquenchable}\right)=\frac{F\left(\infty ,q\right)\cdot \left(1+K_{\text{SV}}\cdot {\left[{\text{Tl}}^{+}\right]}_{e}\right)-F\left(0,b\right)}{K_{\text{SV}}\cdot {\left[{\text{Tl}}^{+}\right]}_{e}}\text{.}$$ (8)

The time course of fluorescence quench thus can be expressed by

$$F\left(t\right)=\frac{F\left(\text{extravesicular}\right)}{1+K_{\text{SV}}\cdot {\left[{\text{Tl}}^{+}\right]}_{e}}+\frac{F\left(\text{intravesicular}\right)}{1+K_{\text{SV}}\cdot {\left[{\text{Tl}}^{+}\right]}_{i}\left(t\right)}+F\left(\text{unquenchable}\right)$$ (9)

or

$$F\left(t\right)=\frac{F\left(\infty ,q\right)\cdot \left(1+K_{\text{SV}}\cdot {\left[{\text{Tl}}^{+}\right]}_{e}\right)-F\left(0,q\right)}{K_{\text{SV}}\cdot {\left[{\text{Tl}}^{+}\right]}_{e}}+\frac{1+K_{\text{SV}}\cdot {\left[{\text{Tl}}^{+}\right]}_{e}}{K_{\text{SV}}\cdot {\left[{\text{Tl}}^{+}\right]}_{e}}\cdot \frac{F\left(0,q\right)-F\left(\infty ,q\right)}{1+K_{\text{SV}}\cdot {\left[{\text{Tl}}^{+}\right]}_{i}\left(t\right)}\text{.}$$ (10)

Inserting *K*_SV_ = 60 M^–1^ (Ingólfsson and Andersen, 2010) and [Tl^+^]_e_ = 25 mM, Eq. 10 reduces to:

$$F\left(t\right)=\frac{2.5\cdot F\left(\infty ,q\right)-F\left(0,q\right)}{1.5}+\frac{2.5}{1.5}\;\cdot \frac{F\left(0,q\right)-F\left(\infty ,q\right)}{1+1.5\cdot \left(1-\exp\left\{1-(1+\frac{t}{\tau_{0}{)}^{\beta }}\right\}\right)}\text{,}$$ (11)

which was fitted to the fluorescence quench traces between 2 ms to 1 s using the non-linear least squares curve fitting method in MATLAB (The MathWorks). Using the resulting values of *F*(0,q), *F*(∞,q), β, and τ_0_, initial rate of fluorescence quench (Tl^+^ influx) could be determined from Eqs. 2 and 11:

$$\begin{array}{l} Rate\left(0\right)={\frac{d}{dt}\left(\frac{F\left(t\right)}{F\left(0,q\right)-F\left(\infty ,q\right)}\right)|}_{0}=-2.5\cdot \frac{\beta }{\tau_{0}}\cdot \\ \frac{exp\left\{1-{\left(1+t/\tau_{0}\right)}^{\beta }\right\}}{{\left\{1+K_{\text{SV}}\cdot {\left[{\text{Tl}}^{+}\right]}_{e}\cdot \left(1-exp\left\{1-{\left(1+t/\tau_{0}\right)}^{\beta }\right\}\right)\right\}}^{2}}|=-2.5\cdot \frac{\beta }{\tau_{0}}\text{.} \end{array}$$ (12)

**Figure 2.**
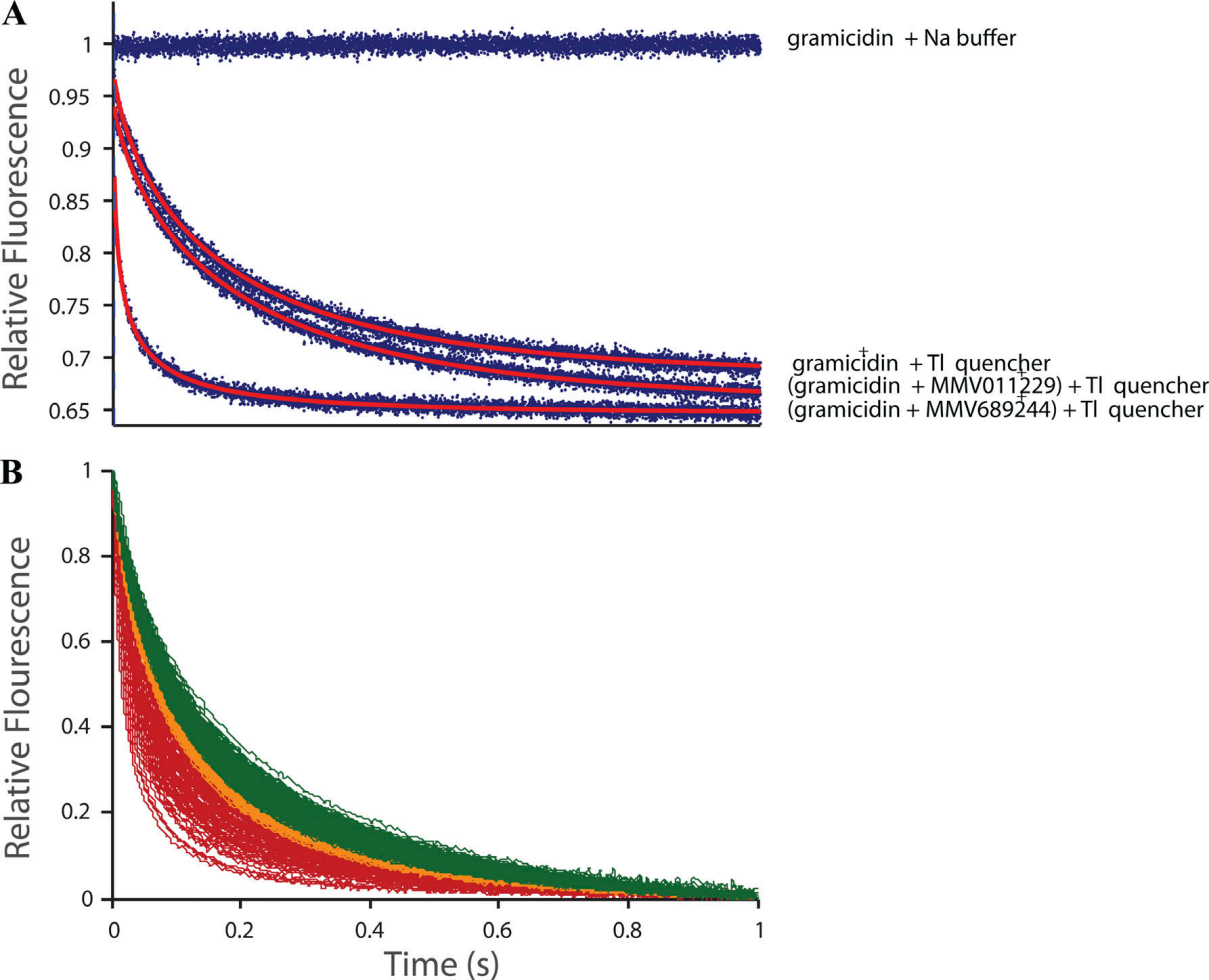
**The effects of the drugs and drug-like molecules on the time course of Tl**^**+**^**-induced quench of ANTS fluorescence. (A)** Results from single mixing reactions for each condition, where the recorded results (blue dots) were fitted by Eq. 11, and the resulting fits are displayed as red curves. The [gramicidin + Na buffer] trace at the top shows the fluorescence of ANTS in the absence of Tl^+^ and drug. The three lower traces show the time course of Tl^+^-induced fluorescence quench in the absence of drug, and the presence of a drug with little bilayer-modifying potency (MMV011229) or a drug with high bilayer-modifying potency (MMV689244). The quench rates were determined by fitting Eqs. 11 and 12 to each curve. In the absence of drug, the rate was 2.9 s^−1^(r^2^ = 0.99). In the presence of 10 µM MMV011229, the rate was 3.1 s^−1^(r^2^ = 0.99); in the presence of 10 µM MMV689244, the rate was 34.6 s^−1^(r^2^ = 0.99). **(B)** The average fluorescence quench traces for all drugs in the Pathogen Box that produced an increase in the fluorescence quench rate. To allow for direct comparison, the traces were normalized by *F*(0,q)–*F*(∞,q), which averaged 0.26 ± 0.05 (mean ± SD). The green traces denote drugs that produce only a modest change (1 ≤ *NormRate* < 1.25; 199 drugs) and therefore have low bilayer-modifying potency; the orange and red traces denote drugs that have moderate (1.25 ≤ *NormRate* < 1.5; 74 drugs) and high (1.5 ≤ *NormRate*; 127 drugs) bilayer-modifying potencies, respectively.

The drug-induced change in quench rate: the quench rate normalized to control, *NormRate*, was determined as follows:

$$NormRate=\frac{Rate}{Rate_{\text{cntrl}}}=\frac{Rate_{\text{drug}}\left(0\right)}{Rate_{\text{cntrl}}\left(0\right)}\text{.}$$ (13)

where the subscripts cntrl and drug denote the rates in the absence and presence of the drug, respectively. For display, e.g., Fig. 2, the traces are normalized to *F*(0,b).

The drug-induced changes in bilayer deformation energy $\Delta \Delta G_{bilayer|drug}^{M\to D}=\Delta G_{bilayer,drug}^{M\to D}-\Delta G_{bilayer,cntrl}^{M\to D}$ were evaluated as (Artigas et al., 2006; Sun et al., 2019; Sun et al., 2020):

$$\begin{array}{l} {\Delta \Delta G_{\text{bilayer}}^{M\to D}|}_{\text{drug}}=-RT\cdot \ln\left\{\frac{K_{\text{drug}}^{M\to D}}{K_{\text{cntrl}}^{M\to D}}\right\}=\\ -RT\cdot ln\left\{\frac{{\left[D\right]}_{\text{drug}}/[M{]}_{\text{drug}}^{2}}{{\left[D\right]}_{\text{cntrl}}/[M{]}_{\text{cntrl}}^{2}}\right\}\approx \\ -RT\cdot ln\left\{\frac{{\left[D\right]}_{\text{drug}}}{{\left[D\right]}_{\text{cntrl}}}\right\}\approx \\ -RT\cdot ln\left\{NormRate\right\}\text{,} \end{array}$$ (14)

where *R* is the gas constant; *T* is the temperature in Kelvin; $K_{\text{cntrl}}^{M\to D}$ and $K_{\text{drug}}^{M\to D}$ are the dimerization constants in the absence and presence of drug; and [D]_cntrl_, [D]_drug_, [M]_cntrl_, and [M]_drug_ are the concentrations of dimer (D) and monomer (M), respectively, in the absence or presence of the drug. (Eq. 14 is valid only when [M]_drug_ ≈ [M]_cntrl_, when the monomer↔dimer equilibrium is biased toward the non-conducting monomers in both the absence and presence of the drug in question, as will be the case in the thick DC_22:1_PC bilayers used in this study.)

Some experiments were performed in the presence of 60 µM BSA, as a proxy for the 10% fetal calf serum that often is added to cell culture media. These experiments were performed as described above, except that the LUVs were incubated for 10 min with either 60 µM BSA or 60 µM BSA plus 10 µM of the drug before testing for activity. For the analysis, the drug-induced changes in rates were evaluated as:

$$NormRate_{\text{BSA}}=\frac{Rate_{\text{drug}+\text{BSA}}\left(0\right)}{Rate_{\text{BSA}}\left(0\right)}\text{.}$$ (15)

#### Pan assay interference compounds (PAINS)

The 400 drugs were evaluated for pan assay interference (Baell and Holloway, 2010) using Badapple (http://pasilla.health.unm.edu/tomcat/badapple/badapple; Yang et al., 2016). Badapple detects patterns of promiscuity, assay interference in high-throughput screens, associated with different molecular scaffolds (Yang et al., 2016) and assigns a promiscuity score (*pScore*), which is a measure of the risk of promiscuity. A molecule may incorporate several different scaffolds, which each may yield a different *pScore*, and we report the largest value over all scaffolds. A *pScore* < 100 denotes that pan assay interference is unlikely; 100 ≤ *pScore* < 300 denotes weak likelihood of promiscuity; and 300 ≤ *pScore* indicates high likelihood of promiscuity (Yang et al., 2016).

#### Quantitative estimate of drug-likeness (QED)

The QED (Bickerton et al., 2012) provides a measure of a molecule’s drug-likeness based on eight molecular properties: molecular mass; ALogP; PSA; number of hydrogen bond donors; number of hydrogen bond acceptors; number of rotatable bonds; number of aromatic rings; and number of structural alerts, or unwanted chemical groups (Brenk et al., 2008). We estimated the drugs’ QED score using the RDKit chemoinformatics software (http://www.rdkit.org/) with average descriptor weights (Bickerton et al., 2012).

#### Cluster analysis

To evaluate how different groups of molecules (grouped based on *NormRate*, or *CC*_20_) clustered, we used silhouette analysis (Rousseeuw, 1987). Silhouette plots visualize how close the objects in a cluster are grouped compared to neighboring clusters. For each molecule in a cluster, a silhouette score is calculated based on the average dissimilarity (distance to other molecules) in the cluster compared to the dissimilarity to molecules in other clusters. The silhouette score ranges between 1 (good separation among clusters) and −1 (poor/non-existent separation among clusters). The scores for all molecules in a cluster are averaged; the higher the average score, the better this cluster is defined.

#### Statistics

The quality of the fits of Eq. 11 to the fluorescence quench traces was judged by the regression coefficient for the fits, (average 0.996 ± 0.015; six samples had regression coefficients below 0.95%, all were >0.70).

All experiments were performed in duplicate, and the results are reported as mean ± range/2. The average range/(2⋅mean) was 0.07 ± 0.07 (mean ± SD); six drugs had range/(2⋅mean) >0.3, they were tested in triplicate and the results are reported as mean ± SD. Comparisons among different groups of drugs were carried out using the two-tailed Mann–Whitney test using the Bonferroni correction for multiple comparisons when needed.

When results are presented as box plots (Fig. S4, S6, S7, S8, S10, and S11), the lengths of the bottom and top whiskers were calculated using the MatLab boxplot function as 1.5⋅(q3 – q1), where q1 and q3 denote the first and third quartiles (in case of a normal distribution, this provides 99.3% coverage of the data between the top and bottom whiskers); values below the bottom and above of the top whisker are defined as outliers and denoted by +, and the whiskers end with the largest (smallest) value that fall within the range defined by the whiskers. *CC*_20_ values in the MMV database were truncated at 80 µM, and some plots may not have a top whisker.

### Online supplemental material

**Figure S3.**
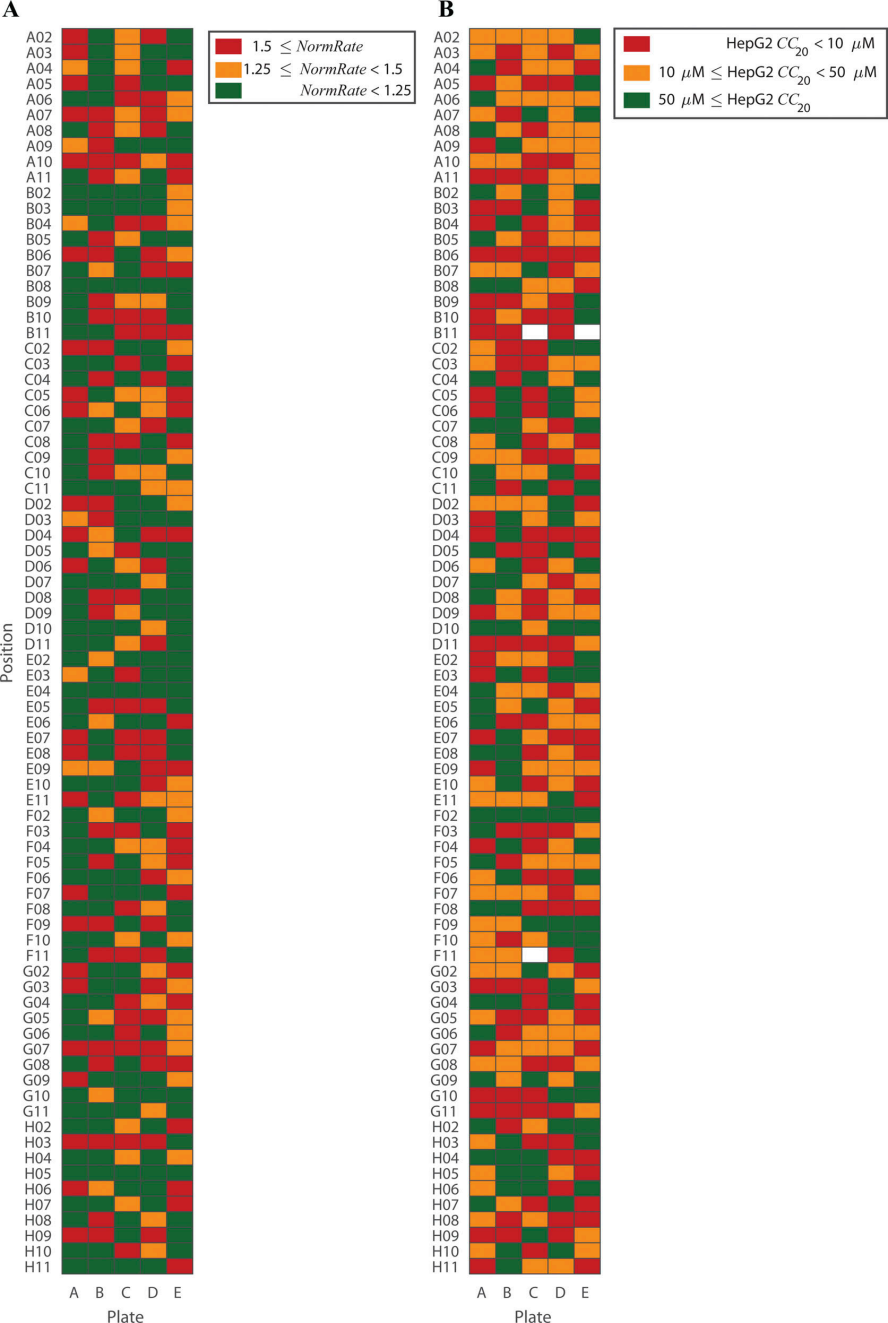
**The distribution of *NormRates* and HepG2 *CC*_20_ with respect to the physical plate mapping of the Pathogen Box.** Each column (A–E) represents a 96-well plate; the location of the drug in each plate (A02–H11) is denoted by the labels to the left. **(A)** Distribution of *NormRates*. Green boxes denote drugs producing modest changes in bilayer properties (*NormRate* ≤ 1.25); orange and red boxes show drugs with moderate (1.25 ≤ *NormRate* < 1.5) and high (1.5 ≤ *NormRate*) bilayer-modifying potencies, respectively. **(B)** Distribution of HepG2 *CC*_20_ values. Green boxes denote drugs with 50 µM ≤ *CC*_20_ (meaning they are relative nontoxic); orange and red boxes denote drugs with 10 µM ≤ *CC*_20_ < 50 µM and *CC*_20_ < 10 µM, respectively (which are likely to be toxic), see also Fig. S5 B; white boxes denote drugs without *CC*_20_ information.

**Figure S4.**
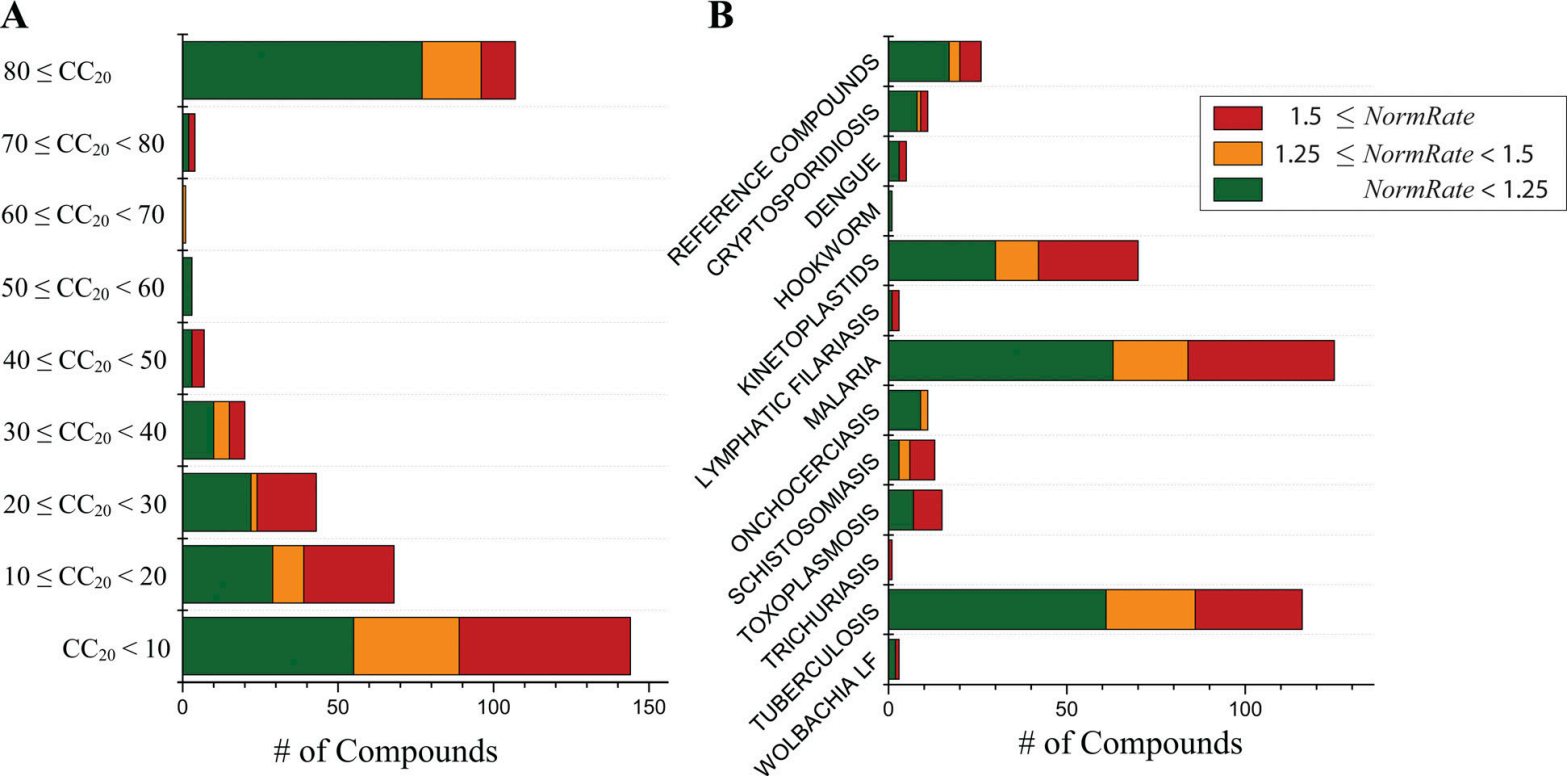
**Distribution of drugs in the Pathogen Box by their HepG2 *CC*_20_ values, *NormRates* and intended disease.** Green denotes bilayer-inert drugs (*NormRate* < 1.25); orange denotes moderately bilayer-modifying drugs (1.25 ≤ *NormRate* < 1.5); and red denotes potent bilayer-modifying drugs (1.5 ≤ *NormRate*). **(A)** Distribution of drugs ranked by their HepG2 *CC*_20_ values (for the 397 drugs where the information is available [MMV]); the likelihood a drug will be bilayer-modifying (1.25 ≤ *NormRate*) increases with decreasing *CC*_20_ values (when the drugs become more likely to be cytotoxic); molecules with *CC*_20_ < 50 µM are likely to have in vivo cytotoxicity (Greene et al., 2010). **(B)** Distribution of drugs by their intended target disease. Less than 50% of the drugs that are active against lymphatic filariasis, schistosomiasis, toxoplasmosis, and trichuriasis are bilayer-inert.

**Figure S5.**
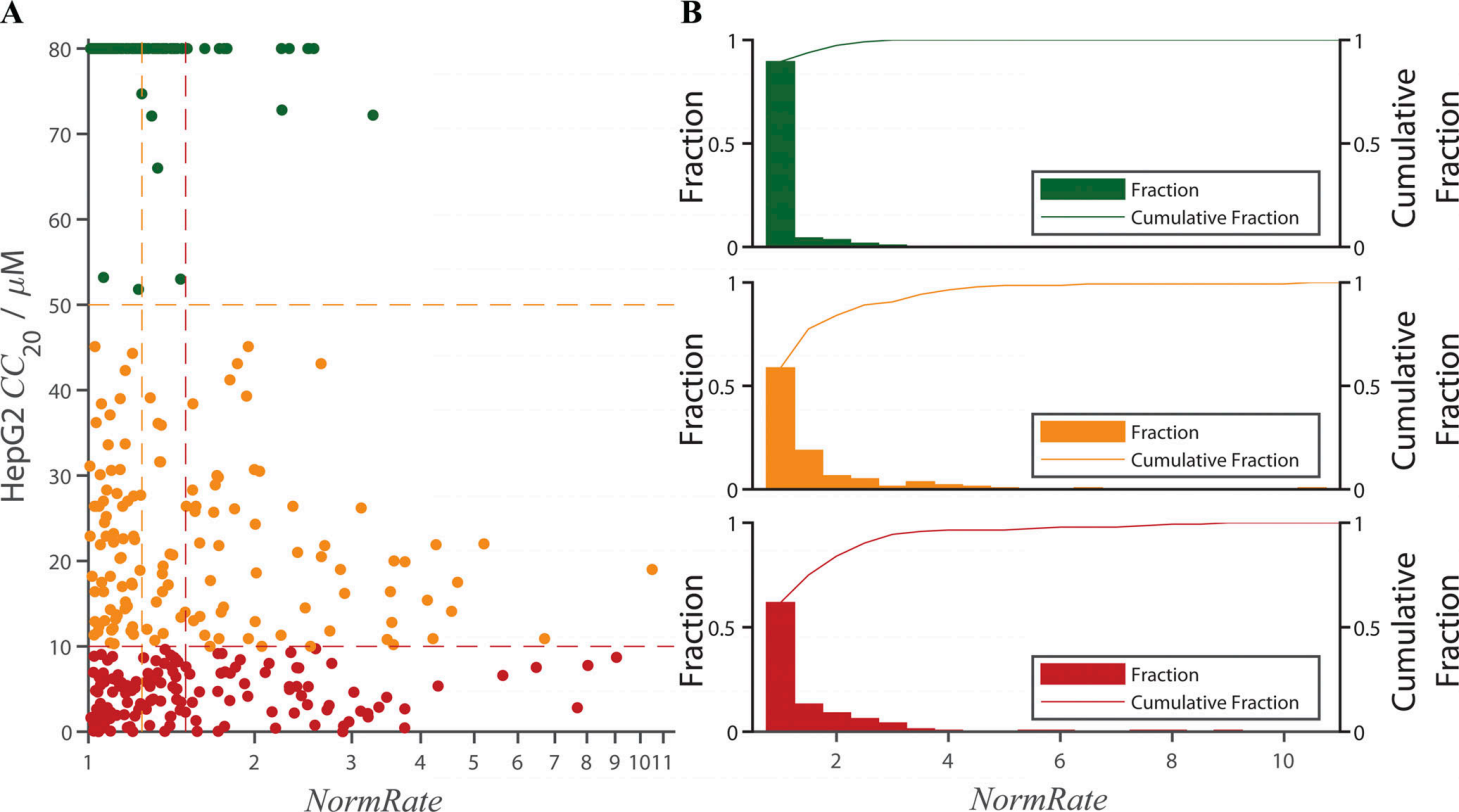
**Distribution of HepG2 *CC*_20_ values (from MMV) as function of *NormRate* ([Drug] = 10 µM; *CC*_20_ values ≥ 80 μM have been set to 80 μM). (A)** Scatter plot of HepG2 *CC*_20_ vs. *NormRate*; green dots denote drugs with 50 µM ≤ *CC*_20_; orange dots denote drugs with 10 µM ≤ *CC*_20_ < 50 µM; red dots denote drugs with *CC*_20_ < 10 µM. There is a weak correlation between *CC*_20_ and *NormRate* (r^2^ < 0.06); correlation line not shown. **(B)** Fractional distribution of *NormRates* for drugs with 50 µM ≤ *CC*_20_ (top green graph), 10 µM ≤ *CC*_20_ < 50 µM (middle orange graph), and *CC*_20_ < 10 µM (bottom red graph); the curves at the top of each graph shows the cumulative distribution. Using ANOVA with Bonferroni correction for multiple comparisons there is a significant difference between the distributions for drugs with 50 µM ≤ *CC*_20_ and drugs with 10 µM ≤ *CC*_20_ < 50 µM (P = 4.8⋅10^–4^) or drugs with *CC*_20_ < 10 µM (P = 1.3⋅10^–4^), as well as between drugs with 50 µM ≤ *CC*_20_ and drugs with *CC*_20_ < 50 µM (P = 6.8⋅10^–8^). There is no difference between the distributions for drugs with 10 µM ≤ *CC*_20_ < 50 µM and drugs with *CC*_20_ < 10 µM (P = 1).

**Figure S6.**
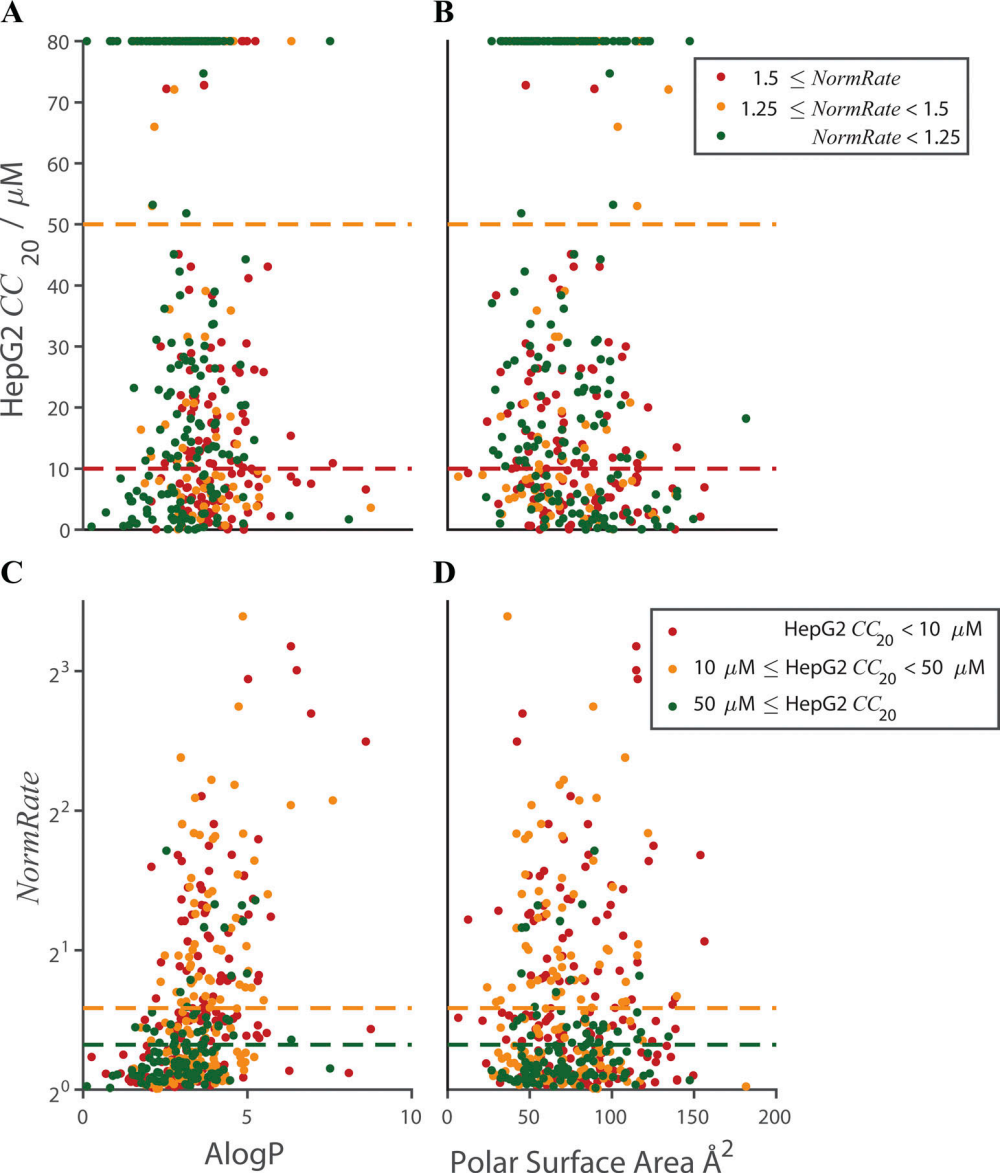
**Scatter plots of the distributions of HepG2 *CC*_20_ and *NormRate* vs. ALogP and PSA. (A and B)** In A and B, HepG2 *CC*_20_ vs. ALogP and PSA; green dots denote drugs with *NormRate* < 1.25; orange dots denote drugs with 1.25 ≤ *NormRate* < 1.5; red dots denote drugs with 1.5 ≤ *NormRate*. The orange and red lines denote the *CC*_20_ thresholds for moderate and high toxicity, respectively. **(C and D)** In C and D, *NormRate* (on base-2 logarithmic scale) vs. ALogP and PSA; green dots denote drugs with 50 µM ≤ *CC*_20_; orange dots denote drugs with 10 µM ≤ *CC*_20_ < 50 µM; red dots denote drugs with *CC*_20_ < 10 µM. The green and orange lines denote the *NormRate* thresholds for moderate and high bilayer perturbation, respectively. There is no correlation between *CC*_20_ and ALogP (r^2^ < 0.05) or PSA (r^2^ < 1.8⋅10^–3^). There is a weak correlation between *NormRate* and ALogP (r^2^ < 0.1), and no correlation between *NormRate* and PSA (r^2^ < 2.6⋅10^–5^). Correlations lines not shown.

**Figure S7.**
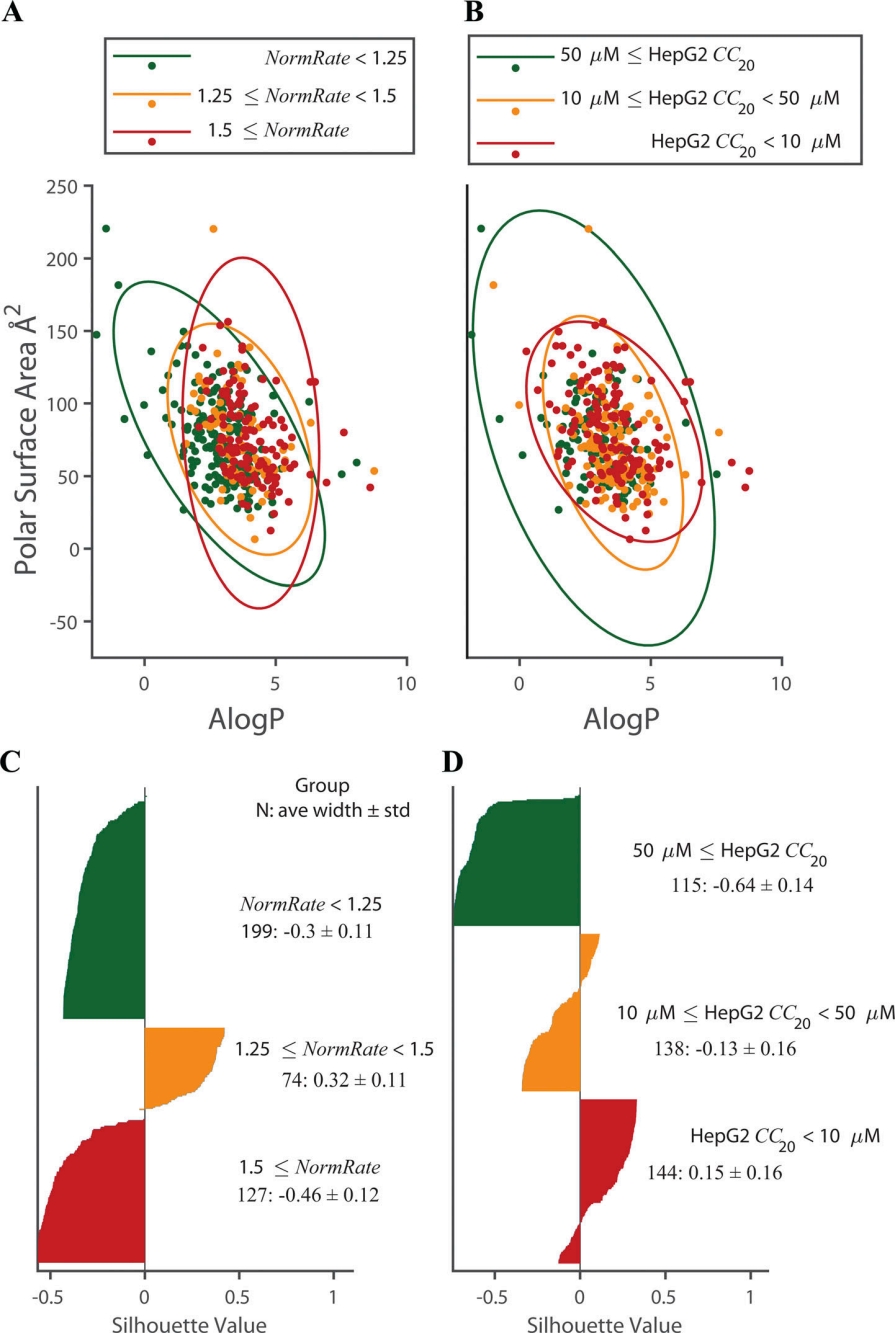
**Bilayer-modifying potency and cytotoxicity as function of ALogP and PSA.** In each panel the drugs are divided into three clusters, as identified in the insets. **(A and B)** In A and B, the three ellipses denote the 95% confidence limits for each cluster, identified by its respective color. **(A)** Green dots denote drugs that cause minimal bilayer perturbation; orange dots are drugs that cause moderate effects on the bilayer; red dots denote potent bilayer-modifying drugs. **(B)** Green dots denote drugs that are relatively nontoxic to the HepG2 cell line; orange dots are drugs that are slightly toxic; red dots are the more potent drugs. **(C)** Silhouette plot (see Materials and methods) for the drugs in A, which allows evaluating the quality of the clustering (the separation among the clusters). The higher the average silhouette score, the better the clusters are separated (Rousseeuw, 1987). When clustering drugs by *NormRates*, the average silhouette width is 0.25 for the 400 drugs. **(D)** Silhouette plot for the drugs in B. When clustering by HepG2 *CC*_20_ s, the average silhouette width is −0.17 for the 397 drugs. Based on the overlap among the three ellipses and the modest/negative silhouette scores, we conclude that ALogP and PSA together are not robust predictors of a given drug’s bilayer-modifying potency or toxicity against the HepG2 cell line.

**Figure S8.**
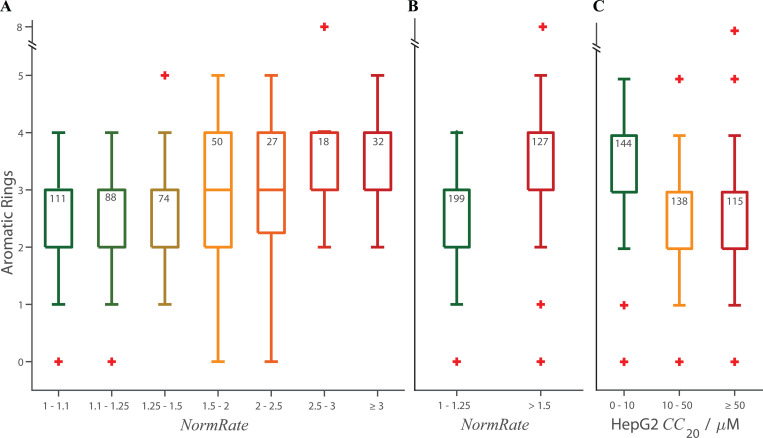
**Box plots of the number of aromatic rings per drug vs. *NormRate*; + denotes outliers, the number of drugs in each group are noted at the top of the box. (A)** The full range of number of aromatic rings per drug vs. *NormRate*. Based on visual inspection, the distribution for drugs with *NormRates* < 1.25 differ from that for drugs with *NormRates* ≥ 2 (P = 7.3·10^−6^ using a two-tailed Mann–Whitney test). **(B)** The distributions of aromatic rings per drug for drugs with *NormRates* < 1.25 differ from that for drugs with 1.5 ≤ *NormRates* (P = 1.5·10^−6^). **(C)** The distribution of aromatic rings per drug for drugs with 50 ≤ *CC*_20_ likewise differs from that for drugs with *CC*_20_ < 10 (P = 2⋅10^−6^).

**Figure S9.**
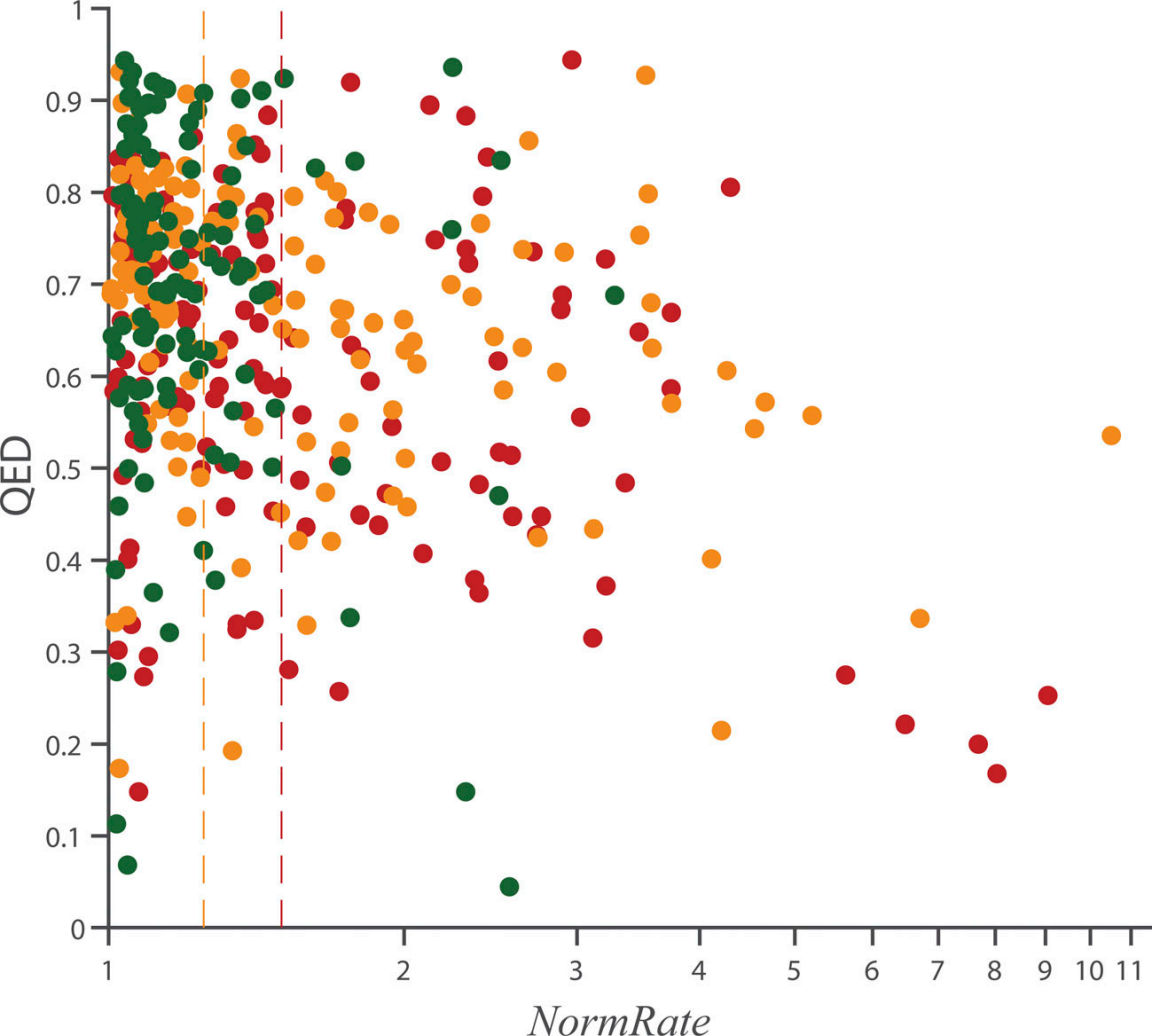
**Scatter plots of the distribution of QED vs. *NormRate* (logarithmic x axis).** Green dots denote drugs with 50 µM ≤ *CC*_20_; orange dots denote drugs with 10 µM ≤ *CC*_20_ < 50 µM; red dots denote drugs with *CC*_20_ < 10 µM. The vertical lines denote *NormRate* = 1.25 and 1.5, respectively. There is no correlation between the two descriptors (r^2^ = 0.08, correlation line not shown).

**Figure S10.**
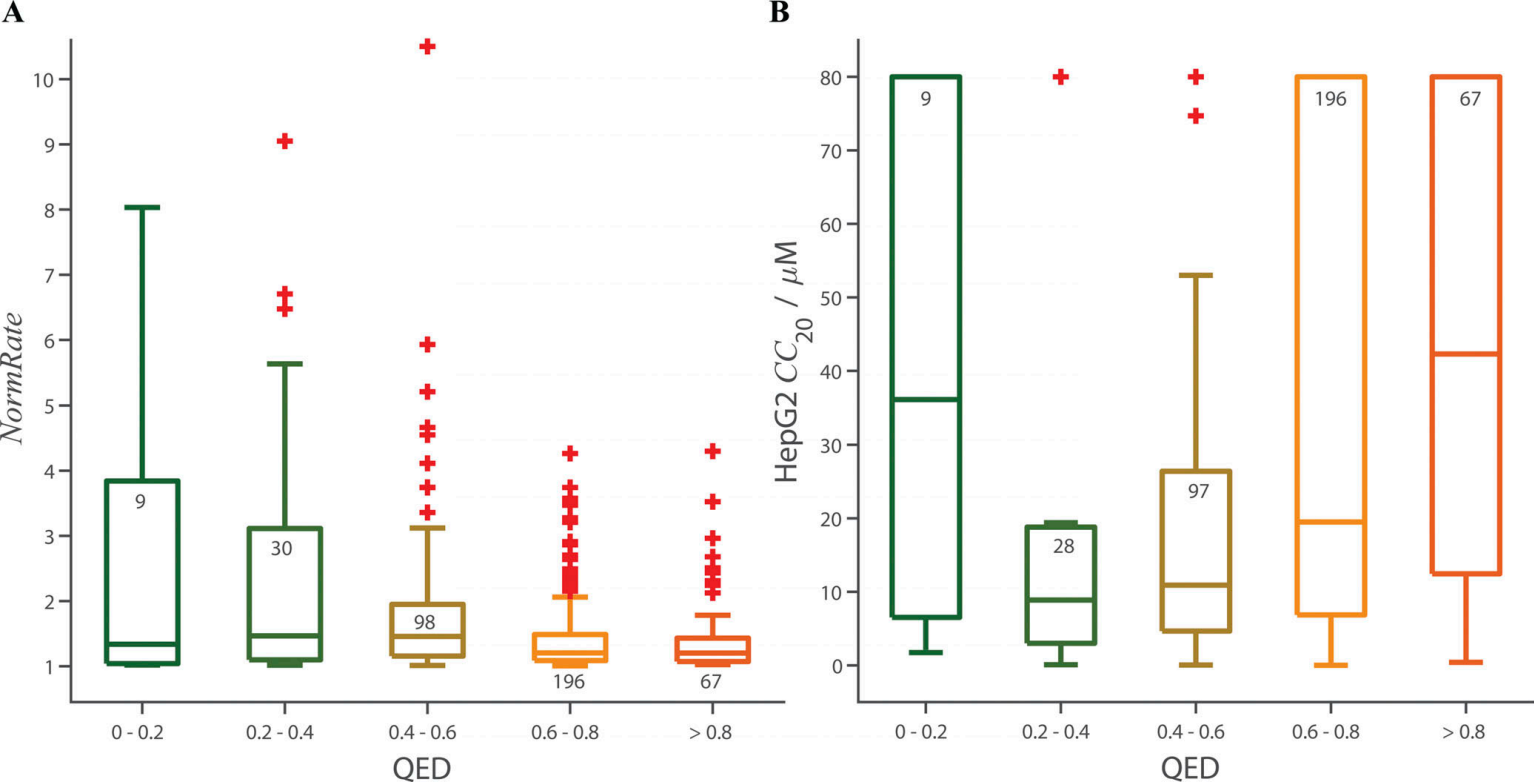
**Box plots of the distribution of *NormRates* and HepG2 *CC*_20_ values as functions of the drugs’ QED scores; + denotes outliers, the number of drugs in each group are noted at the top of the box. (A)** Results for *NormRate*, comparing the groups with QED ≤ 0.4 and 0.6 < QED there is no significant difference between the *NormRates* (P = 0.06). **(B)**. Results for *CC*_20_, comparing the groups with QED ≤ 0.4 and 0.6 < QED there is again no significant difference between the *CC*_20_ values (P = 0.06).

**Figure S11.**
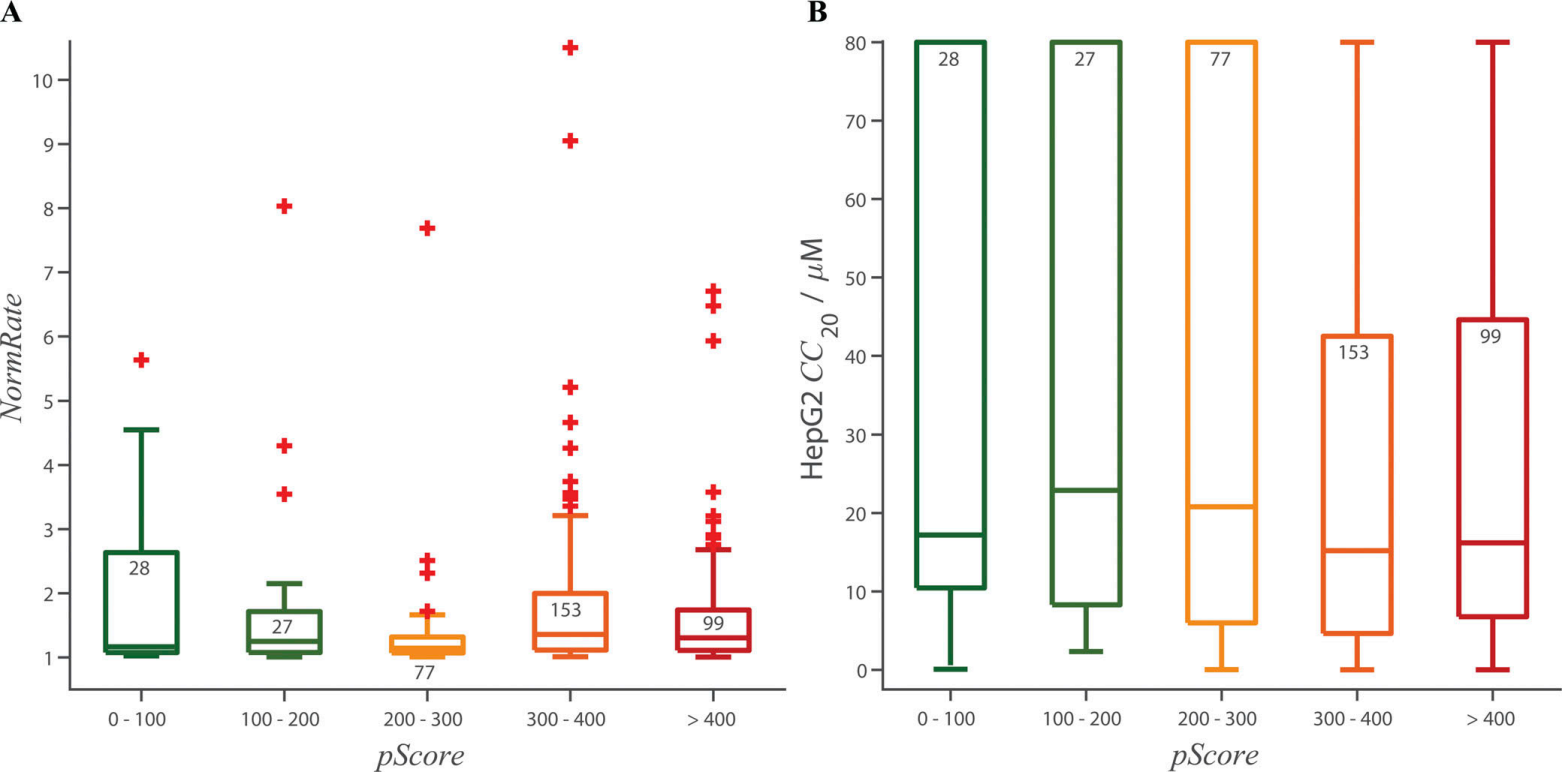
**Box plots of the distribution of *NormRates* and HepG2 *CC*_20_ concentrations as functions of pScore; + denotes outliers, the number of drugs in each group are written at the top of each box. (A)** Results for *NormRate*; comparing the groups with *pScore* < 200 and *pScore* ≥ 300, there is no significant difference between the two groups (P = 0.45). **(B)** Results for *CC*_20_; comparing the groups with *pScore* < 200 and *pScore* ≥ 300, drugs with higher *pScore* are more likely to have low *CC*_20_ values (P = 0.03).

Fig. S1 shows gramicidin channel structure and function. Fig. S2 shows the distribution of LUV diameters for vesicles used in the fluorescence quench experiments. Fig. S3 shows distribution of *NormRates* and HepG2 *CC*_20_ among drugs in the Pathogen Box. Fig. S4 shows distribution of drugs in the Pathogen Box by their intended disease, quench rates, and HepG2 *CC*_20_. Fig. S5 shows the distribution of HepG2 *CC*_20_ values (from MMV) as function of *NormRate*. Fig. S6 shows scatter plots of the distributions of HepG2 *CC*_20_ and *NormRate* vs. ALogP and PSA. Fig. S7 shows bilayer-modifying potency and cytotoxicity vs. ALogP and PSA. Fig. S8 shows distribution of aromatic rings per drug vs. *NormRate*. Fig. S9 shows scatter plot of the distribution of QED vs. *NormRate*. Fig. S10 shows box plots of the distribution of *NormRates* and HepG2 *CC*_20_ as function of QED. Fig. S11 shows box plots of the distribution of *NormRates* and HepG2 *CC*_20_ as function of *pScore*. Table S1 provides detailed information about the 400 drugs in the Pathogen Box, which lists HepG2 *CC*_20_ values (for 397 drugs), information on protein binding (fumouse and fumic), *NormRates* and Range or SD, estimates of the drug concentrations in the aqueous and membrane phases, QED (along with the eight properties used to calculate it), and the *pScore*. Tables S2, S3, S4, and S5 provide effect of albumin on bilayer-modifying potency; average ALogP and PSA for drugs with low, intermediate, or high bilayer-modifying potency or cytotoxicity; odds for drugs being bilayer-modifying or cytotoxic vs. ALogP; and chemically similar drugs in the Pathogen Box, respectively. Three text sections are provided at the end of the PDF.

## Results and discussion

We first present results on the bilayer-modifying effects of the drugs in the Pathogen Box, as evaluated using stopped-flow fluorescence quench experiments to quantify their effects on the gramicidin monomer↔dimer equilibrium. We then consider the bilayer-mediated regulation of membrane protein function, emphasizing that the drugs tested here alter lipid bilayer properties as opposed to disrupting the membrane barrier properties. Next, we consider the question of the drug concentrations in the membrane required to cause the changes in quench rate and cytotoxicity (evaluated as changes in HepG2 *CC*_20_) and the relation(s) between molecular structure and bilayer-modifying potency. Then, we consider the possible relationship between bilayer-modifying drugs and PAINS. Finally, we discuss the implications for the use of small molecules to manipulate biological function including drug development.

### Stopped-flow fluorescence quench experiments reveal correlation between bilayer-modifying potency and cytotoxicity

The molecules’ bilayer-modifying potency was examined with 10 µM drug in the system (aqueous plus membrane phase) using stopped-flow spectrofluorometry.

Fig. 2 A shows fluorescence quench traces recorded with fluorophore-loaded, gramicidin-doped LUVs that had been equilibrated for 10 min in the absence or presence of drug and then mixed with either buffer or Tl^+^, a gramicidin channel-permeant fluorescence quencher. The quench rate varies with the number of open channels and therefore reflects shifts in the gramicidin monomer↔dimer equilibrium resulting from changes in bilayer properties. For drugs with low bilayer-modifying potency (meaning they produce little change in the monomer↔dimer equilibrium), such as MMV011229, the fluorescence quench traces will be similar to the control traces (absence of drug). For drugs with high bilayer-modifying potency (large shift in the gramicidin monomer↔dimer equilibrium), the fluorescence quench traces will differ from the control traces. MMV0689244, for example, increases the quench rate, which means that Tl^+^ enters the LUVs faster than in the absence of drug because there are more conducting channels in the LUV membrane (the monomer↔dimer equilibrium is shifted toward the conducting dimers).

Fig. 2 B shows the distribution of quench traces recorded in the presence of drugs. Different drugs increase the number of dimers (*NormRate* ≥ 1) to varying extents reflecting their bilayer modifying potency: green, orange, and red traces denote drugs with low (1 ≤ *NormRate* < 1.25), moderate (1.25 ≤ *NormRate* < 1.5), and high (1.5 ≤ *NormRate*) bilayer-modifying potencies. Importantly, the tested drugs did not compromise bilayer integrity—increase leakage of intravesicular contents during the 10 min incubation, which would result in instantaneous quench when drug-treated LUVs were exposed to the Tl^+^ quencher. This was not observed for any drug. Leakage of the trivalent ANTS out of the LUVs is slow, meaning undetectable after 24 h at 25°C: the initial drop in fluorescence, evaluated as *F*(0,b), is 0.02 ± 0.03 after 10 min and 0.08 ± 0.05 after 24 h under control conditions. In the presence of 5% ethanol, the positive control used in these studies, it was 0.16 ± 0.03 after 10 min and 0.17 ± 0.03 after 24 h; in the presence of 200 µM CHAPS, it was 0.09 ± 0.03 after 10 min and 0.09 ± 0.01 after 24 h.

The quench traces were fit with Eq. 11, the initial rates of Tl^+^ influx at t = 0 (Rate(0)) were calculated using Eq. 12, and the bilayer-mediated shift in the monomer↔dimer equilibrium was estimated as the *NormRate*, the quench rate in the presence of the drug normalized to the rate in the absence of drug, Eq. 13. The resulting *NormRates* are listed in Table S1, which also includes detailed information about the 400 drugs in the Pathogen Box.

**Figure 3.**
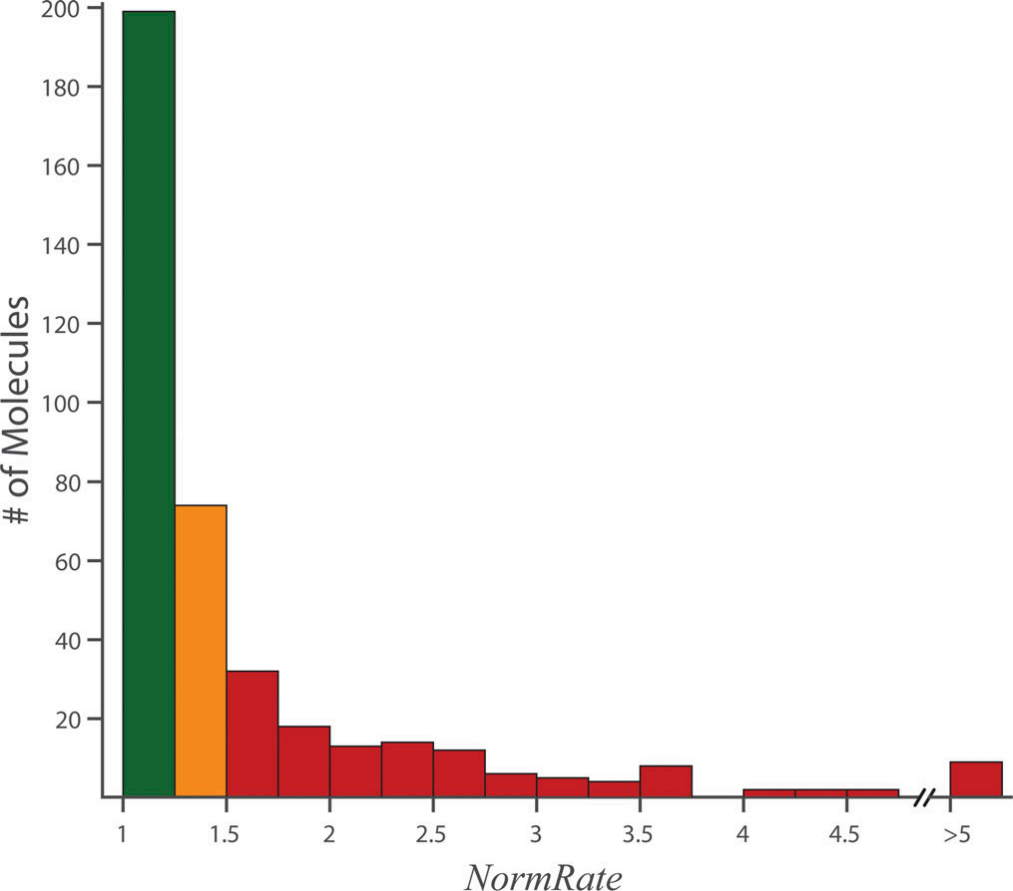
**Distribution of *NormRates* for drugs in the Pathogen Box.** Green bins denote drugs with a modest change in bilayer properties (1 ≤ *NormRate* < 1.25); orange and red bins denote drugs with moderate (1.25 ≤ *NormRate* < 1.5) and high (1.5 ≤ *NormRate*) bilayer-modifying potencies, respectively. Unless otherwise noted, this color code will be used in all figures.

Fig. 3 shows the distribution of *NormRates* binned by increasing *NormRates* for the 400 drugs in the Pathogen Box (for drugs with *NormRate* < 1, the results are plotted as 1/*NormRate*); see also Fig. S3 A and Fig. S4 A. Fig. S4 B shows the distribution of *NormRates* among drugs with different intended target diseases; there is little difference among the groups.

Comparing the drugs’ bilayer-modifying potency to their likely cytotoxicity, reported by MMV as HepG2 *CC*_20_ values (MMV, 2017), there was weak correlation between the two (r^2^ < 0.06) when analyzed using a simple scatter plot (Fig. S5 A). Drugs with high *NormRates* tend to have low *CC*_20_ values, but drugs with low *NormRates* do not necessarily have high *CC*_20_ values.

**Figure 4.**
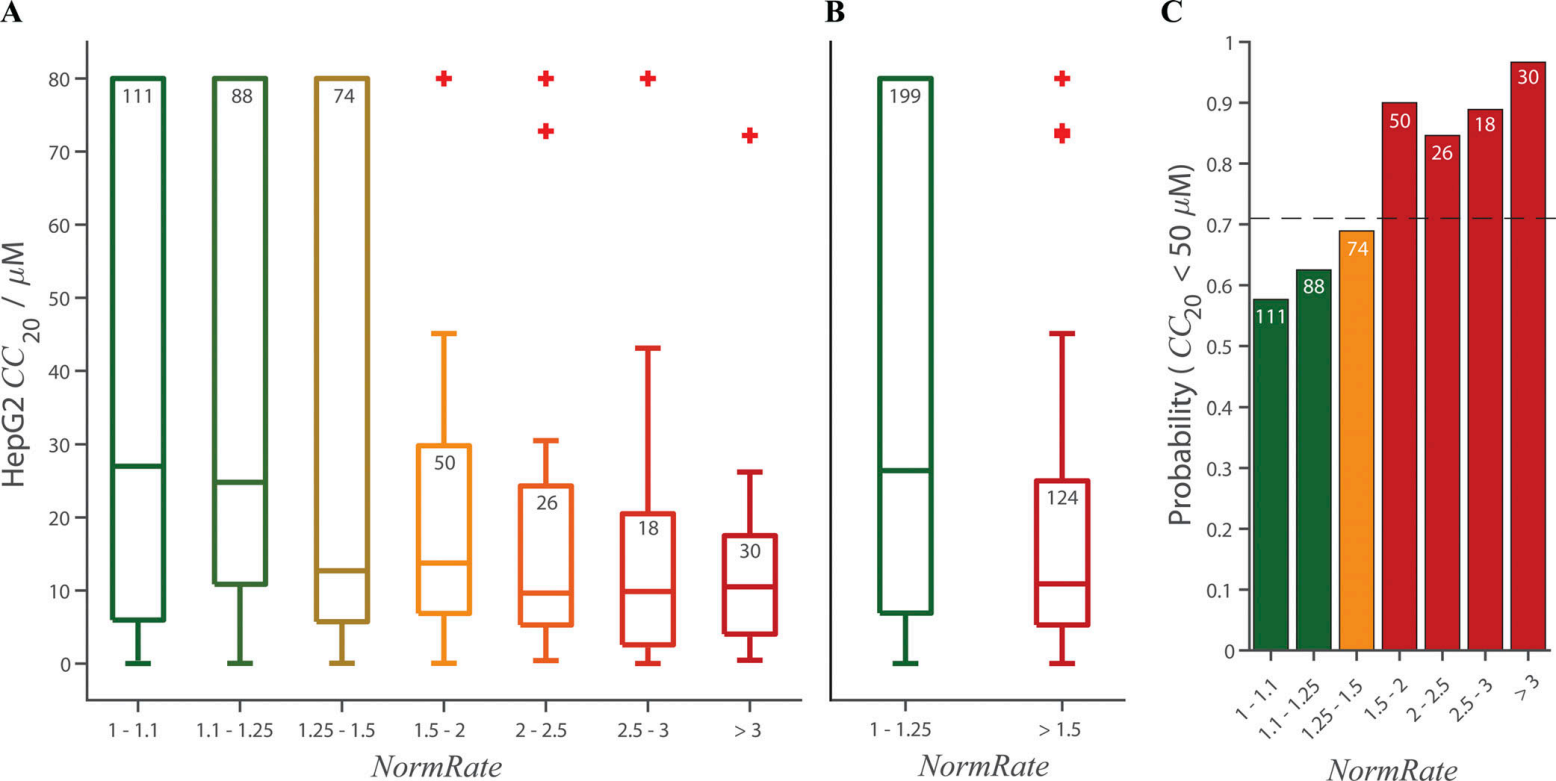
**Box plots of the distributions of HepG2 *CC***_**20**_ **values, from MMV, as function of *NormRate*.** [Drug] = 10 µM; + denotes outliers in the groups (see Statistics in Materials and methods); the number of drugs in each group are noted at the top of the box, and a + may indicate more than one drug. (*CC*_20_ values ≥ 80 μM were set to 80 μM by MMV). **(A)** 50% of the drugs increase *NormRate* by <25% and have a wide distribution of *CC*_20_ values. 31% of the drugs increase *NormRate* by >50% and have a narrower distribution of *CC*_20_ values. **(B)** Comparing the HepG2 *CC*_20_ distributions for drugs with 1 ≤ *NormRate* < 1.25 and drugs with 1.5 *≤ NormRate* the difference is significant (using a two-sided Mann–Whitney test, P = 2.1·10^−6^). Drugs with intermediate *NormRates* (1.25 ≤ *NormRate* < 1.5) have a wide distribution of *CC*_20_ values, similar to drugs with *NormRate* < 1.25, but an average *CC*_20_ closer to that for drugs with *NormRate* ≥ 1.5. Comparing drugs with 1.25 ≤ *NormRate* < 1.5 to those with 1 ≤ *NormRate* < 1.25 or those with 1.5 ≤ *NormRate*, the distribution of HepG2 *CC*_20_ values for drugs with 1.25 ≤ *NormRate* < 1.5 is closer to the distribution for drugs with 1.5 ≤ *NormRate* (P = 0.7) than to the distribution for drugs with 1 ≤ *NormRate* < 1.25 (P = 0.06). **(C)** Probability that a drug has a *CC*_20_ < 50 µM as function of *NormRate*. The stippled horizontal line denotes the average probability a drug has a *CC*_20_ ≤ 50 µM (= 0.71).

Binning the results by *NormRate* (Fig. 4), we found that more potent bilayer modifiers (those producing larger changes in *NormRate*) tend to have lower *CC*_20_ values (more likely to be cytotoxic; e.g., Greene et al., 2010).

Fig. 4 A shows the distribution of *CC*_20_ values binned by increasing *NormRates*. By inspection, drugs with 1 ≤ *NormRate* < 1.25 tend to have higher HepG2 *CC*_20_ values than drugs with 1.5 ≤ *NormRate*. Comparing the HepG2 *CC*_20_ distributions for the 199 drugs with 1 ≤ *NormRate* < 1.25 and the 124 drugs with 1.5 ≤ *NormRate*, the difference is significant, P = 2.1 · 10^–6^ (Fig. 4 B). As expected from Fig. 4 A, the probability a drug has a *CC*_20_ <50 µM (and therefore is likely to be cytotoxic; Greene et al., 2010) increases with increasing *NormRate* (Fig. 4 C); 90% of drugs with 1.5 ≤ *NormRate* have *CC*_20_ < 50 µM (100% of drugs with *NormRate* ≥ 4), whereas only 60% of drugs with *NormRate* < 1.25 have *CC*_20_ < 50 µM. Changes in quench rate therefore do not provide a rule to determine whether or not a molecule will be cytotoxic, rather they provide a measure of the probability that a molecule may be cytotoxic.

When comparing the distributions of *NormRates* for different ranges of HepG2 *CC*_20_ values (Fig. S5 B), there is a significant difference between drugs with 50 µM ≤ *CC*_20_ and drugs with *CC*_20_ < 50 µM (P = 6.8 ⋅ 10^–8^). Overall, Figs. 4 and S5 show that high quench rates tend to be associated with low *CC*_20_ values, meaning that drugs that are potent bilayer modifiers tend to be cytotoxic. The opposite need not be true, drugs with low quench rates may have a significant probability of being cytotoxic because drugs may be cytotoxic for reasons that are unrelated to the membrane. It is in this context relevant that the drugs in the Pathogen Box by design are likely to be cytotoxic (at least, for their intended target) through mechanisms that may not involve the bilayer.

Some drugs with 1.5 ≤ *NormRate* have large *CC*_20_ values (some are marked as outliers, denoted by +, in Fig. 4). This could be due to binding to proteins in the cell culture medium used in cytotoxicity assays or to metabolism, which would reduce the free concentrations (and likelihood of cytotoxicity). We retested these drugs in the presence of 60 µM BSA (Table S2). In all cases, *NormRate* in the presence of BSA (NormRateBSA) was less than *NormRate* in the absence of BSA, suggesting that these drugs (except, maybe, MMV688330) indeed bind to albumin. This was confirmed by independent information about the fraction of unbound drugs (*fu*) in the presence of *fu*_mic_, *fu*_mouse_ (MMV, 2017). Other tested drugs may also bind to protein; we do not consider this further.

### Potent bilayer modifiers tend to alter membrane protein and cell function

The results in Figs. 4 and S5 show that the extent of drug-induced changes in bilayer properties (quantified as changes in *NormRate*) allow for predicting the likelihood a drug or drug-lead will be cytotoxic: 90% of drugs with 1.5 ≤ *NormRate* have *CC*_20_ < 50 µM (100% of drugs with 4 ≤ *NormRate*). Changes in cell membrane composition and lipid bilayer physical properties have long been known to alter membrane protein function (Seeman, 1972; Sandermann et al., 1978; Spector and Yorek, 1985; Bienvenüe and Marie, 1994; Andersen, 2007), which in turn will alter cell function (Spector and Yorek, 1985) and, when the changes in cell function are of sufficient magnitude, may cause cytotoxicity. Changes in protein function arise because the conformational equilibria of transmembrane transporters, channels, and receptors are sensitive to changes in their lipid bilayer environment. In some cases, e.g., the phosphoinositides (Hilgemann et al., 2018; Thompson and Baenziger, 2020; Cheng et al., 2022), specific lipid molecules function as direct or allosteric modulators of membrane protein function. In other cases, the regulation is due to the changes in lipid bilayer physical properties like thickness, intrinsic curvature, and the associated elastic moduli (Brown, 1994; Andersen, 2007).

**Figure 5.**
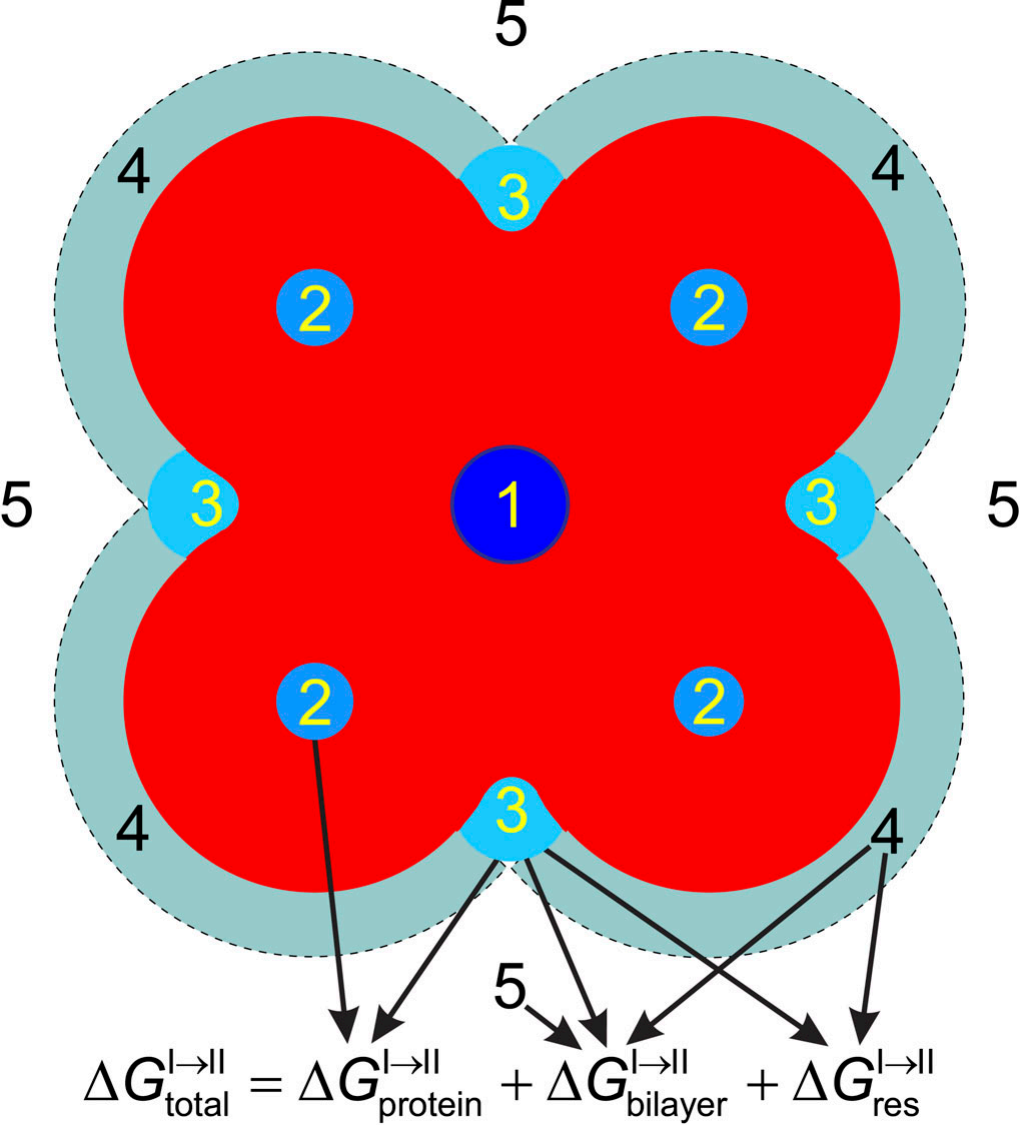
**Schematic ion channel with potential sites for drug modulation of function and the associated contributions to** $\Delta G_{\mathrm{total}}^{I\to \mathrm{II}}\text{.}$ 1, binding in the pore (active site) to block ion movement, which may be small, maybe zero; 2, binding to allosteric sites formed by the protein, which will contribute to $\Delta G_{\text{protein}}^{I\to \text{II}}$ (e.g., Jackson, 1989); 3, binding to allosteric sites formed by the protein plus bilayer lipids, which will contribute both $\Delta G_{\text{protein}}^{I\to \text{II}}$ and $\Delta G_{\text{bilayer}}^{I\to \text{II}}$ (e.g., Obi and Natesan, 2022), as well as the so-called residual exposure contribution $\left(\Delta G_{\mathrm{res}}^{I\to \mathrm{II}}\right)$ due to imperfects hydrophobic matching between membrane proteins and their host bilayer (Mondal et al., 2011); 4, non-specific binding/enrichment at the protein/bilayer boundary, which will contribute to $\Delta G_{\text{bilayer}}^{I\to \text{II}}$ and maybe also $\Delta G_{\text{res}}^{I\to \text{II}};$ and 5, partitioning into the bilayer/solution interface to alter bulk bilayer properties, which will contribute to $\Delta G_{\text{bilayer}}^{I\to \text{II}}.$ After Andersen, 2008 and Herold et al., 2014; see also Urban, 2002.

As suggested by Spector and Yorek (1985), using different language, the changes in protein function are likely to reflect, at least in part, changes in the energetic cost of the bilayer adaptation/deformation to membrane protein conformational changes, which is the bilayer contribution (Δ*G*_bilayer_) to the free energy cost of the conformational changes (Δ*G*_total_) that underlie protein function (see Section 1 at the end of the PDF). The changes in cell function, in turn, reflect the aggregate result of indiscriminate changes in the function of many different membrane proteins that support membrane transport and cell signaling functions. The structure of, and conformational changes in, membrane proteins are, of course, different from the transmembrane dimerization of (nearly cylindrical) subunits, and the bilayer-mediated regulation of integral membrane proteins is likely to have features not observed with gramicidin channels, though conformational changes in transporters involve changes in the local lipid packing (Norimatsu et al., 2017; Wang and Boudker, 2020) that may be associated with substantial changes in Δ*G*_bilayer_ (Zhou et al., 2019). Fig. 5 shows a highly schematized membrane protein (ion channel) highlighting different non-exclusive mechanisms for drug modulation of membrane protein function (see also Payandeh and Volgraf, 2021).

The mechanisms range from binding to the pore/active site to block ion movement/catalytic activity (Site 1), over allosteric regulation arising from (specific) binding to the target protein (Sites 2 and 3), to allosteric regulation that arises from (less specific) drug-induced changes in lipid structure and dynamics in the lipid shells adjacent to the protein (Sites 3 and 4), and in bulk bilayer material properties (Site 5). For any protein, $\Delta G_{def}^{I},$ $\Delta G_{def}^{II},$ and $\Delta G_{\text{bilayer}}^{I\to \text{II}}$ vary with bilayer thickness, intrinsic lipid curvature and the associated elastic moduli, which are determined by intermolecular interactions among the membrane lipids (Helfrich, 1981; Venable et al., 2015); Δ*G*_*def*_ also depends on protein “shape,” protein-bilayer hydrophobic mismatch that reflect interactions between embedded proteins and their adjacent lipids (Cantor, 1997; Dan and Safran, 1998; Mondal et al., 2011; Corradi et al., 2019; Zhou et al., 2019; Obi and Natesan, 2022).

The lipid bilayer-dependent parameters pertain to all membrane proteins, whether gramicidin channels or integral membrane proteins, meaning that changes in gramicidin channel function will predict changes in integral membrane protein function (Lundbaek et al., 2005; Rusinova et al., 2011; see also Lundbaek et al., 2010a), though the magnitude of the drug-induced changes in channel function (*NormRate*) varies with bilayer composition (Elliott et al., 1985; Bruno et al., 2007; Rusinova et al., 2011; Rusinova et al., 2015; Herold et al., 2017; Sun et al., 2020), which could reflect changes in drug partition coefficients (e.g., Haydon et al., 1977; Mason et al., 1992), or that the bilayer contribution to the free energy of dimer formation varies with lipid composition (Sun et al., 2020). The protein-specific parameters depend on the protein in question, meaning that the bilayer-mediated regulation of protein function by small molecules may include contributions that are not detected by gramicidin channels (e.g., Rusinova et al., 2021).

In the case of orally administered drugs, drug-target engagement (Hughes et al., 2011; Simon et al., 2013; Stefaniak and Huber, 2020) requires that drugs cross one or more membranes, whether by solubility-diffusion through the membranes’ lipid bilayer component or by protein-catalyzed mechanisms (Sugano et al., 2010; Smith et al., 2014; Basore et al., 2015). Irrespective of the mechanism by which they cross cell membranes, many drugs and drug leads are sufficiently hydrophobic that they partition into the membranes’ bilayer/solution interface (e.g., Seeman, 1972; Avdeef et al., 1998; Rusinova et al., 2011; Kapoor et al., 2019; Bennett et al., 2020), where they will alter many, if not all, bilayer properties including thickness, intrinsic curvature, acyl chain order, elasticity, fluidity, phase transition temperature, and others (e.g., Seddon et al., 2009). Changes in any of these properties may impact membrane protein function through their aggregate effect on the bilayer contribution, $\Delta G_{\text{bilayer}}^{I\to \text{II}},$ to the free energy cost of membrane protein conformational changes, with the dominant mechanism likely to be the thermodynamic membrane softening caused by the reversible partitioning of drugs into the bilayer/solution interface (Evans et al., 1995; Zhelev, 1998; Lundbaek et al., 2010b; Rusinova et al., 2011; Kapoor et al., 2019). Though the term “fluidity” often is invoked, A. G. Lee showed long ago (Lee, 1991) that changes in fluidity do not serve as a primary mechanism for regulating membrane protein function. The accumulation of drugs into the bilayer/solution interface may also alter protein function by mechanisms that involve more direct, if non-specific, interactions (Andersen, 2008; Payandeh and Volgraf, 2021; Rusinova et al., 2021; Cheng et al., 2022), which also would alter the free energy cost of protein conformational changes, or cause even more complex changes such as phospholipid hydrolysis (Baciu et al., 2006), phospholipidosis (Tummino et al., 2021), or changes in membrane domain organization (Fricke et al., 2022).

Importantly, the changes in $\Delta G_{\text{bilayer}}^{I\to \text{II}}$ (and the ensuing changes in protein and cell function that may cause cytotoxicity) occur at drug concentrations that do not cause a breakdown in membrane barrier properties (as evident from the fluorescence quench traces in Fig. 2). Rather, the drugs cause more subtle changes in bilayer properties that corrupt normal membrane protein function, and thereby cell homeostasis and signaling.

### Drug concentrations in the membrane is a factor in cytotoxicity

The drugs in the Pathogen Box have been tested for cytotoxicity on HEPG2 cells and reported as HepG2 *CC*_20_ values (MMV, 2017), which we used for our evaluation of the relation between bilayer-modifying potency and likely cytotoxicity. Previous studies have shown that ∼95% of molecules tested for cytotoxicity have similar effects on different cell lines (Lin and Will, 2012; Chiaravalli and Glickman, 2017; Lee et al., 2020).

We tested for bilayer-modifying effects at 10 µM, a concentration commonly used to determine a molecule’s cytotoxicity (Chiaravalli and Glickman, 2017). Our results can be extrapolated to other concentrations because a bilayer-modifying molecule’s effect on bilayer properties, as estimated using the gramicidin monomer↔dimer equilibrium, varies as an approximately linear function of the aqueous drug concentration (Ingólfsson and Andersen, 2010; Ingólfsson and Andersen, 2011; Alejo et al., 2013; Kapoor et al., 2019), which allows for estimating the bilayer-modifying effect (change in *NormRate*) at other drug concentrations (see Section 3 and Eq. S2 at the end of the PDF).

The relevant concentrations in this context are the actual drug concentrations in the membrane ([Drug]_m_) and the aqueous phase ([Drug]_a_), where [Drug]_a_ may be less than the nominal concentration in the system, [Drug]_nom_, because drugs will equilibrate between the aqueous and membrane phases (Bruno et al., 2007; Rusinova et al., 2011; Heerklotz and Keller, 2013; Kapoor et al., 2019). Knowing a drug’s partition coefficient, *K*_1_, defined as *K*_1_ = [Drug]_m_/[Drug]_a_, [Drug]_a_ and [Drug]_m_ can be expressed as:

$${\left[\text{Drug}\right]}_{a}=\frac{{\left[\text{Drug}\right]}_{\text{nom}}}{1+K_{1}\cdot V_{\text{lip}}/V_{\text{aq}}};{\left[\text{Drug}\right]}_{m}=\frac{K_{1}\cdot {\left[\text{Drug}\right]}_{\text{nom}}}{1+K_{1}\cdot V_{\text{lip}}/V_{\text{aq}}}\text{,}$$ (16)

where *V*_lip_ and *V*_aq_ denote the volumes of the lipid (acyl chains plus head group) and aqueous phases, respectively (*V*_lip_/*V*_aq_ = 3.6 ⋅ 10^–5^ in our experiments; see Section 3 and Eqs. S22 and S23 at the end of the PDF). Approximating K_1_ by ALogP, we can estimate [Drug]_a_ and [Drug]_m_ at [Drug]_nom_ = 10 µM. These [Drug]_a_ and [Drug]_m_ estimates are summarized in Table S1; the [Drug]_a_ estimates vary between 0.5 nM (for the most hydrophobic drugs) and 10 µM, the associated [Drug]_m_ estimates vary between sub-pM (for the least hydrophobic drugs) and 280 mM. In any case, it will be important to evaluate a drug candidate’s bilayer-modifying potency at the actual, unbound concentrations where it has its desired effects. Drugs that exert their on-target effects at low nanomolar concentrations may well be bilayer modifying (and cytotoxic) at 10 μM, but that may not to be important at the concentrations where the drugs exert their desired effects.

### Physicochemical properties are weak predictors of bilayer-modifying potency and cytotoxicity (HepG2 *CC*_20_)

Drug candidates’ physicochemical properties are important for their successful development (e.g., Lipinski et al., 2001; Leeson and Springthorpe, 2007; Hughes et al., 2008; Bickerton et al., 2012). Physicochemical properties determine not only a drug’s pharmacokinetic profile (Lipinski et al., 2001) but also its promiscuity (Leeson and Springthorpe, 2007), toxicity (Hughes et al., 2008), and overall drug-likeness (Bickerton et al., 2012). Yet, standard physico-chemical properties (ALogP and PSA) do not allow for robust prediction of cytotoxicity.

Fig. 4, plus Fig. S4 A and Fig. S5, shows that the tested drugs’ bilayer-modifying potency is correlated with their cytotoxicity (as quantified by their HepG2 *CC*_20_). In contrast, there was little/no correlation between cytotoxicity and physicochemical parameters used in drug design (Hughes et al., 2008; Price et al., 2009) such as ALogP (r^2^ < 0.05) and PSA (r^2^ < 1.8⋅10^–3^; Fig. S6). Fig. S6, A and B, shows the distribution of HepG2 *CC*_20_ values vs. ALogP and PSA, respectively. Fig. S6, C and D, show the corresponding distribution of *NormRates* vs. ALogP and PSA. There is a weak correlation between the drugs’ *NormRate* and ALogP (r^2^ < 0.1), but no correlation with PSA (r^2^ < 2.6⋅10^–5^); Table S3 summarizes information about the average ALogP and PSA values for the six groups (low, medium, and high *NormRate* or *CC*_20_). Focusing on ALogP, comparing drugs with ALogP ≤ 3 and ALogP > 3, the odds ratios for a drug being bilayer-modifying or cytotoxic are 3.8 and 2.0, respectively, (95% CI: 2.4–5.9) and (1.3–3.1); Table S4.

Yet, both ALogP and PSA are likely to be important for a drug’s partitioning in the bilayer/solution interface. Combining ALogP and PSA did not yield strong correlation with our experimental results (Fig. S7). But following Hughes et al. (2008) (see also Price et al., 2009), who found that molecules with CLogP > 3 with a relatively small total polar surface area (TPSA < 75 Å2) were more likely to be promiscuous and cytotoxic, we calculated the odds for a drug being bilayer-modifying (*NormRate* ≥ 1.25) after grouping the drugs into four groups: ALogP ≤ 3 (low ALogP) and > 3 (high ALogP); PSA ≤ 75 Å2 (low PSA) and > 75 Å2 (high PSA; Table 1, top). The bottom of Table 1 shows the corresponding information for drugs having *CC*_20_ < 50 µM.

**Table 1.** Odds for drugs being bilayer active or likely cytotoxic vs. ALogP/PSA

	Odds for drugs having 1.25 ≤ *NormRate*/*NormRate* < 1.25	
	PSA ≤ 75 Å^2^	PSA > 75 Å^2^
ALogP ≤ 3	6/38 = 0.16	32/61 = 0.52
ALogP > 3	99/72 = 1.38	58/34 = 1.71
	**Odds for drugs having ** $\frac{{\mathrm{CC}}_{20}<50µM}{50µM\leq {\mathrm{CC}}_{20}}$	
	PSA ≤ 75 Å^2^	PSA > 75 Å^2^
ALogP ≤ 3	25/19 = 1.32	59/34 = 1.74
ALogP > 3	129/41 = 3.15	69/21 = 3.29

Top: The odds ratio for a drug being bilayer-modifying (having 1.25 ≤ *NormRate*) is 11-fold higher (95% CI: 4.1–28.2) for drugs with ALogP > 3 and PSA > 75 Å^2^, relative to drugs with ALogP ≤ 3 and PSA ≤ 75 Å^2^. Bottom: The odds ratio for a drug being cytotoxic (having HepG2 CC_20_ < 50 µM) is 2.5-fold higher (95% CI: 1.1–5.6) for drugs with ALogP > 3 and PSA > 75 Å^2^, relative to drugs with ALogP ≤ 3 and PSA ≤ 75 Å^2^. We only have HepG2 CC_20_ information for 397 of the 400 drugs in the Pathogen Box.

Bilayer-modifying potency and the likelihood that a drug is cytotoxic (has a *CC*_20_ < 50 µM) increase with increasing ALogP and, for a given ALogP range, the bilayer-modifying potency and cytotoxicity (though to a lesser extent) increase with increasing PSA. Whereas neither ALogP nor PSA are robust predictors of bilayer-modifying potency or likely cytotoxicity (Table S3), the combination of a high ALogP (≥3) and PSA (≥75 Å^2^) is associated with increased bilayer-modifying potency: comparing drugs having low ALogP and PSA with drugs having high ALogP and PSA, the odds ratio for drugs having a *NormRate* ≥1.25 is 11-fold higher for the latter group (Table 1).

Drugs with high ALogP and PSA, which will tend to localize near the bilayer/solution interface, thus tend to be more potent bilayer modifiers than drugs with low ALogP and PSA. Yet, despite the odds ratios, ALogP together with PSA do not allow for predicting a drug’s bilayer-modifying potency because of overlap among the groups (Fig. S7). For cytotoxicity: comparing drugs having ALogP ≤ 3 and PSA ≤ 75 Å^2^ to drugs having ALogP > 3 and PSA > 75 Å^2^, the odds ratio for a drug having a HepG2 *CC*_20_ < 50 µM is only 2.5-fold higher for the latter group, which may be due to the high number of cytotoxic drugs with modest bilayer-modifying potency (drugs may be toxic for reasons that have nothing to do with the membrane). In contrast to Hughes et al. (2008), however, for drugs with ALogP > 3, those with PSA > 75 Å^2^ are as likely to be cytotoxic (have *CC*_20_ < 50 µM) as those with PSA ≤ 75 Å^2^.

We also explored other parameters commonly considered including molecular mass, number of heavy atoms, number of hydrogen bond donors or acceptors, and number of Rule of 5 violations (Lipinski et al., 2001); none showed meaningful correlation to *NormRate* (r^2^ < 0.1, r^2^ < 0.1, r^2^ < 8.8⋅10^–6^, r^2^ < 3.9⋅10^–3^, and r^2^ < 0.1, respectively) or HepG2 *CC*_20_ (r^2^ < 6.3⋅10^–3^, r^2^ < 6.8⋅10^–3^, r^2^ < 4.6⋅10^–5^, r^2^ < 2.1⋅10^–3^, and r^2^ < 4.6⋅10^–3^, respectively). There was a correlation with the number of aromatic rings in the drugs (Fig. S8); drugs with more aromatic rings tended to have higher *NormRates*, which may reflect that molecules with more aromatic rings are likely to be more hydrophobic (e.g., Ritchie and Macdonald, 2009); the average ALogP of drugs with ≤2 aromatic rings was 3.0, whereas the average ALogP of drugs with ≥3 aromatic rings was 3.7.

The above parameters (plus information about structural alerts; Brenk et al., 2008) have been combined into the QED score (Bickerton et al., 2012), which is a weighted score based on the following descriptors: molecular mass, ALogP, PSA, number of hydrogen bond donors, number of hydrogen bond acceptors, number of rotatable bonds, number of aromatic rings, and number of structural alert. Although QED was not developed to predict toxicity per se, successful drugs cannot possess unacceptable toxicity, meaning that QED scores implicitly include an assessment of toxicity. Fig. S9 shows the distribution of QED vs. *NormRate* (r^2^ < 0.08).

**Figure 6.**
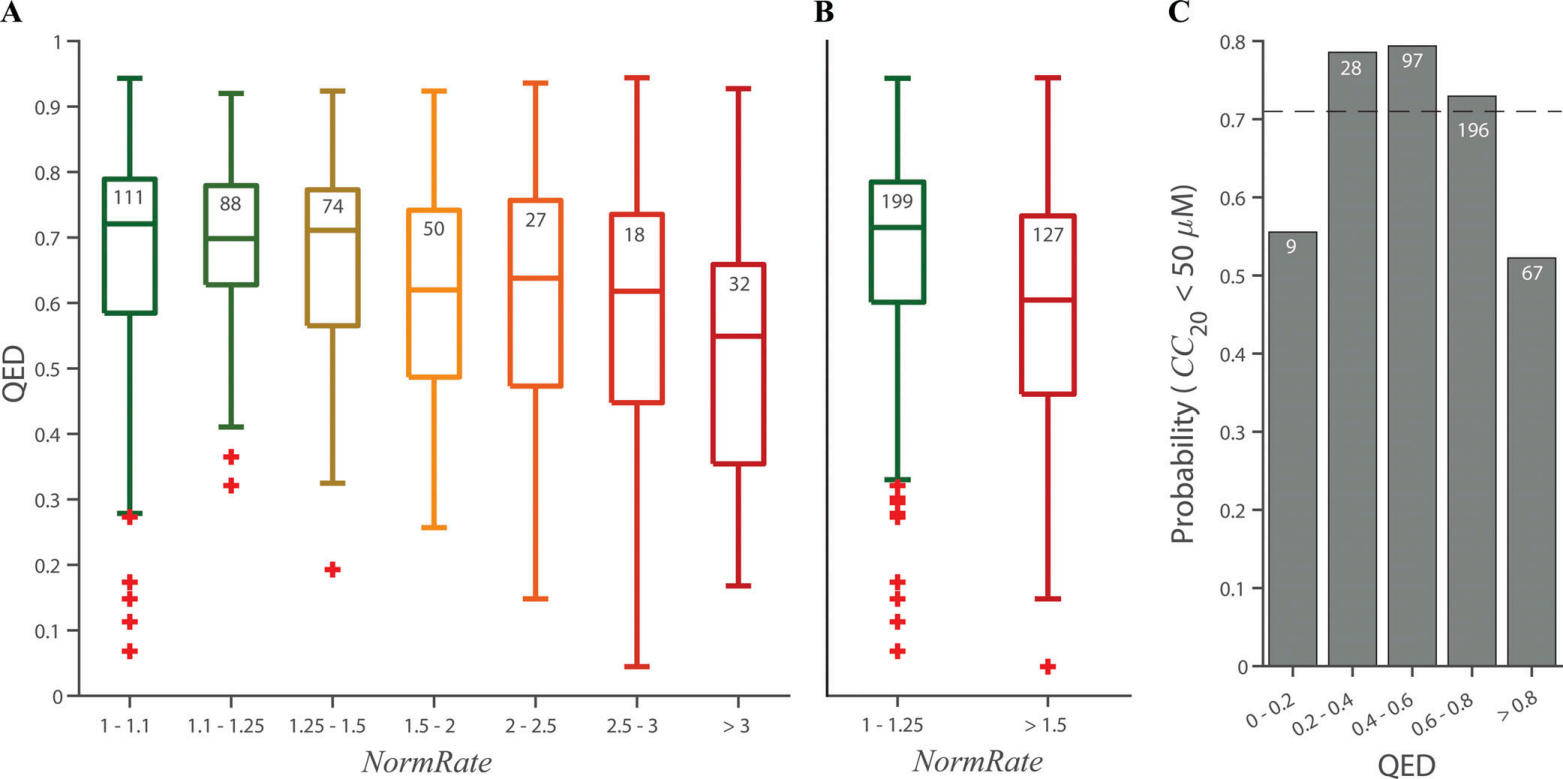
**Box plots of the distribution of QED as function of *NormRate*; + denotes outliers in the groups, the number of drugs in each group are noted at the top of the box. (A)** QED decreases with increasing *NormRat*e. **(B)** There is a significant difference between the distributions of QED values for *NormRate* < 1.25 and 1.5 ≤ *NormRate* (P = 4.2⋅10^−7^). **(C)**. Probability that a molecule has a *CC*_20_ < 50 µM as a function of QED and the stippled horizontal line denotes the average probability a drug has a *CC*_20_ ≤ 50 µM (= 0.71).

Fig. 6 shows the binned distribution of QED values as function of *NormRate*. QED tends to decrease with increasing *NormRate* (Fig. 6 A). Comparing drugs with *NormRate* < 1.25 and 1.5 ≤ *NormRate*, (Fig. 6 B) high *NormRates* tend to be correlated with low QED scores (P = 4.2⋅10^–7^). Fig. S10 shows the relation between QED and *NormRate* or *CC*_20_ and QED; there is no systematic trend in either distribution or the probability that a drug has a *CC*_20_ < 50 µM does not vary significantly between molecules with 0 ≤ QED < 0.2 and 0.8 ≤ QED ≤ 1 (Fig. 6 C).

Combining *NormRate* and QED, however, provides improved prediction of drugs having *CC*_20_ < 50 µM: 82% of drugs with *NormRate ≥* 1.25 have *CC*_20_ < 50 µM (*n* = 198); 79% of drugs with QED < 0.5 have *CC*_20_ < 50 (*n* = 71), whereas 89% of drugs with *NormRate* ≥ 1.25 and QED < 0.5 have *CC*_20_ < 50 (*n* = 47).

### Bilayer-modifying potency does not predict and pan assay interference

**Figure 7.**
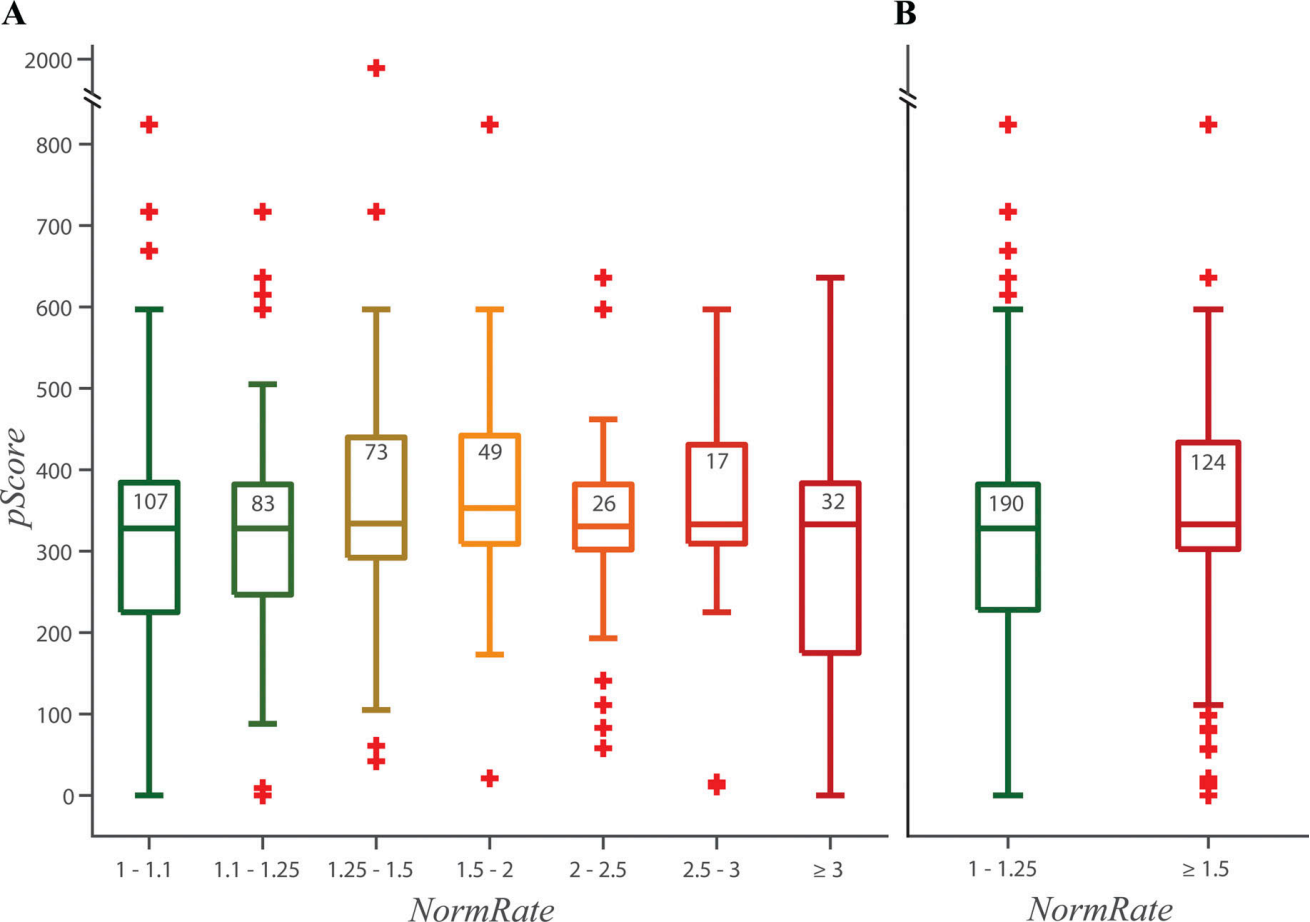
**Box plots of the distribution of *pScores* as function of *NormRate*; + denotes outliers in the groups, the number of drugs in each group are noted at the top of the box. (A)** The distribution of *pScores* varies little with *NormRate*. **(B)** Comparing the distribution of *pScores* for 1 ≤ *NormRate* < 1.25 and 1.5 ≤ *NormRate* using the two-tailed Mann–Whitney test; there is no difference between the two groups (P = 0.51).

A perennial problem in drug development is the so-called PAINS (Baell and Holloway, 2010) or nuisance compounds (Dahlin et al., 2021), and some PAINS are potent bilayer modifiers (Baell and Walters, 2014; Aldrich et al., 2017). Yet, the molecular properties that cause a molecule to be bilayer-active (hydrophobicity, sufficient polarity to localize to the bilayer/solution interface) do not involve chemical reactivity, and bilayer-modifying potency per se may not be sufficient to cause a molecule to be promiscuous and masquerade as a hit in high-throughput protein-based screens. We, therefore, explored the relationship between a drug’s bilayer-modifying potency and its promiscuity index (*pScore*) using Badapple (http://datascience.unm.edu/public-biocomputing-apps; Yang et al., 2016). The results (Fig. 7, A and B; and Fig. S11 A) show that there is no correlation between bilayer-modifying potency, quantified as *NormRate*, and *pScore*, although drugs with high *pScores* are more likely to have low *CC*_20_ values than drugs with low *pScores* (Fig. S11 B).

Bilayer-active molecules, however, are likely to masquerade as hits in cell-based screens because changes in bilayer properties, measured as changes in the gramicidin monomer↔dimer equilibrium, will produce changes in the function of many, diverse membrane proteins (Lundbaek et al., 2010a; Ingólfsson et al., 2014), which may alter overall cell function, thereby making the molecule appear to be a hit. Bilayer-active molecules thus can be considered as cell-based assay interference compounds (CAINS; see also Sun et al., 2020) or membrane PAINS (Magalhães et al., 2022).

### Drug-induced changes in bilayer properties, implications for drug development and laboratory research?

The motivation for this study was to explore whether it would be possible to use simple and fast biophysical measurements to gain information about the likelihood that a drug candidate will be cytotoxic. Our results show that drug-induced changes in bilayer properties (quantified as *NormRate*) predict the probability a molecule will be cytotoxic, as quantified by its HepG2 *CC*_20_ (Fig. 4). This does not, however, provide a rule for determining whether a molecule is cytotoxic because molecules may be cytotoxic for many reasons that do not involve the bilayer, rather it provides an estimate of the probability a molecule may be cytotoxic, information that will be valuable when interpreting the results of cell physiological experiments of selecting molecules for drug development. This strategy is almost uniquely suited for such a complementary analysis because it is robust and fast: it can be completed (drug equilibration with fluorophore-loaded LUVs, conducting the stopped-flow experiments, and analyzing the results) within 30 min.

The correlation between drug-induced changes in the gramicidin monomer↔dimer equilibrium (lipid bilayer properties) and the changes in HepG2 *CC*_20_, as a measure of cytotoxicity (Fig. 4), show that drug-induced changes in bilayer properties will perturb membrane protein and cell function, which may cause off-target effects and, if the changes in function are large, cytotoxicity. It cannot be excluded, however, that subtle, bilayer-mediated changes in the function of many proteins could produce desired changes in system function (e.g., Eger et al., 2008; Rusinova et al., 2015). In any case, this provides a guide for drug development because chemical modifications that reduce the likelihood of bilayer perturbation, while leaving desired therapeutic effects intact, may produce candidates for development where measurable bilayer perturbation at concentrations much higher than the desired effects (Rusinova et al., 2011; Rusinova et al., 2015)—assuming the desired effect is not due to bilayer-mediated regulation. Substituting the naphthalene (octanol/water partition coefficient = 2.2⋅103; Leo et al., 1971) in propranolol with indole (octanol/water partition coefficient = 1.8⋅102; Leo et al., 1971) in pindolol, for example, reduces the bilayer-modifying potency by an order of magnitude (Rusinova et al., 2015), and the bilayer-modifying potency for short-chain n-alcohols scales with their octanol/water partition coefficients (Ingólfsson and Andersen, 2011). These and other studies (Rusinova et al., 2011; Howery et al., 2012; Dockendorff et al., 2018; Zhang et al., 2018; Kapoor et al., 2019) have shown how seemingly modest alterations in a molecule’s structure—including cis-trans isomerization (Howery et al., 2012)—may produce large changes in its bilayer-modifying potency and effect on membrane protein function. Not surprisingly, therefore, it is possible to modify a potent bilayer-modifying molecule (e.g., resveratrol; Ingólfsson et al., 2014), to minimize its bilayer-perturbing effects while maintaining its desired biological effects (Bosquesi et al., 2020). To our knowledge, this is the first example of a drug candidate that was developed with explicit consideration of its bilayer-modifying properties; a similar strategy has been used to minimize the off-target effects of photostabilizers used to protect fluorescent reporter groups (Grenier et al., 2022).

Table S5 lists pairs of drugs in the Pathogen Box that are chemically similar. Table S5 A lists drugs with different bilayer-modifying potencies; Table S5 B lists drugs with comparable bilayer-modifying potencies. In some cases, the different bilayer-modifying potencies may reflect the drugs’ different hydrophobicity (ALogP) and partitioning into the bilayer; in other cases, e.g., MMV676269 and MMV676270, the differences may reflect drug-induced alterations in the acyl chain dynamics. These results, together with previous studies summarized above, suggest that it may be possible to use a drug-lead’s bilayer-modifying potency as a guide to synthesize analogs that retain the desired biological effects but with less bilayer-modifying potency (see also Payandeh and Volgraf, 2021).

We finally note that the experimental strategy used here also can be used to determine whether changes in membrane protein, or cell, function caused by a bioactive molecule might be due to drug-induced changes in bilayer properties, as opposed to specific, on-target interactions. Platelet-activating factor (1-O-alkyl-2-acetyl-sn-glycero-3-phosphocholine; PAF), for example, binds to a GPCR (Honda et al., 1991) and activates pathways involved in coagulation and inflammation at low nanomolar concentrations (Demopoulos et al., 1979). At high nanomolar concentrations, PAF has additional effects, such as inducing differentiation of cultured neurons, and it becomes cytotoxic at low micromolar concentrations (Kornecki and Ehrlich, 1988). At these concentrations, PAF also alters gramicidin channel function and disrupts bilayer properties (Sawyer and Andersen, 1989) suggesting that bilayer-mediated mechanisms may be involved. Amiodarone is widely used to treat complex cardiac arrythmias (Mujović et al., 2020), but has a complex therapeutic profile and exerts its effects through mechanisms that involve diverse ion channels, transporters, and receptors (Heijman et al., 2013) with no well-defined primary target, suggesting a bilayer-mediated mechanism. Indeed, amiodarone alters bilayer properties at concentrations where it exerts its clinical effects (Rusinova et al., 2015), which may provide a mechanism for its poly-pharmacology (see also Lundbaek et al., 2010a).

At the other extreme, a bilayer-mediated mechanism can be largely excluded if a drug’s desired effects occur at concentrations where it does not appear to alter bilayer properties, as reflected in changes in $\Delta G_{\text{bilayer}}^{M\to D},$ e.g., in the case of general anesthetics (Herold et al., 2014; Herold et al., 2017) or alkylphenol propofol analogs (Tibbs et al., 2013), or there is no correlation between a drug’s effect on its target and its bilayer-modifying potency, e.g., in the case of the marine toxin 6-bromo-2-marcaptotryptamine dimer (BrMT; Dockendorff et al., 2018). It is in this context important that Δ*G*_bilayer_ is the *difference* between two Δ*G*_*def*_s: $\Delta G_{\text{bilayer}}^{M\to D}=\Delta G_{def}^{D}-2\cdot \Delta G_{def}^{M},$ and $\Delta G_{\text{bilayer}}^{I\to \text{II}}=\Delta G_{def}^{\text{II}}-\Delta G_{def}^{I}.$ Drug-induced changes in Δ*G*_bilayer_ thus may be 0 even though the drug changes the underlying Δ*G*_*def*_ s; that is, $\Delta G_{\text{bilayer}}^{I\to \text{II}}=0$ when the changes in $\Delta G_{\mathrm{def}}^{II}$ are equal to the changes in $\Delta G_{\mathrm{def}}^{I}.$

### Conclusions

We have shown that drug-induced changes in the transmembrane gramicidin monomer↔dimer equilibrium in a model membrane composed of a single lipid species correlate with changes in cell function, specifically the drugs’ cytotoxicity, estimated as HepG2 *CC*_20_. Even modest changes in bilayer properties (evaluated as changes in the fluorescence quench rate, which reflect shifts in the gramicidin monomer↔dimer equilibrium) are associated with a reduction in *CC*_20_, indicative of increased risk of cytotoxicity (Fig. 4). Although gramicidin monomer↔dimer transitions differ from conformational transitions in integral membrane proteins, both involve rearrangements within the bilayer hydrophobic core (e.g., Lundbæk et al., 2010a). Drug-induced changes in lipid bilayer properties therefore will alter the distribution among membrane protein conformations, and drug-induced changes in gramicidin channel function can be related to changes in membrane protein function (Lundbaek et al., 2005; Søgaard et al., 2006; Chisari et al., 2010; Lundbaek et al., 2010a; Rusinova et al., 2011; Herold et al., 2014; Ingólfsson et al., 2014; Herold et al., 2017). Because drugs that alter bilayer properties will produce indiscriminate changes in membrane protein and, in turn, cell function, potent bilayer-modifying drugs would be expected to be cytotoxic, at some concentration. The ability to predict changes in cell function based on a simple biophysical measurement shows that the cell membrane’s lipid bilayer moiety is a target for bioactive molecule. It further provides information about the concentrations where a drug can be used to manipulate membrane protein and cell function with minimal risk of bilayer-mediated regulation. Drugs (drug candidates) that exert their desired effects only at concentrations where they modify lipid bilayer properties are unlikely to be successful. The ability to identify such molecules therefore opens up for new, mechanism-based approaches to guide drug development, which may help reduce the cost of developing therapeutics, including drugs for NTD.

## Supplementary Material

Table S1 presents detailed information about the 400 drugs in the Pathogen Box

Table S2 shows on the effect of albumin on bilayer-modifying potency of seemingly non-toxic highly bilayer-modifying drugs

Table S3 shows on the average values for ALogP and PSA for drugs with low, intermediate, or high bilayer-modifying potency or cytotoxicity

Table S4 shows on the odds for drugs being bilayer-modifying or cytotoxic vs. ALogP

Table S5 shows the chemically similar drugs in the Pathogen Box
