# Supplementary material for: Screening for bilayer-active and likely cytotoxic molecules reveals bilayer-mediated regulation of cell function
Source: J Gen Physiol. 2023 Feb 10;155(4):e202213247. doi: 10.1085/jgp.202213247 (PMC9948646; doi:10.1085/jgp.202213247)
Supplement: Table S2 — shows on the effect of albumin on bilayer-modifying potency of seemingly non-toxic highly bilayer-modifying drugs [file JGP_202213247_TableS2.docx]

**Table S2**: Effect of albumin on bilayer–modifying potency
of seemingly non–toxic highly bilayer–modifying drugs.

| Drug | ALogP | PSA / (Å^2^) | HepG2 *CC*_20_(µM) | *NormRate* | *NormRate*_BSA_ | *fu*_mic_ | *fu*_mouse_ |
| --- | --- | --- | --- | --- | --- | --- | --- |
| MMV000016 | 4.30 | 41.67 | > 80 | 2.2 ± 0.3 | 1.7 ± 0.2 | 0.011 |  |
| MMV001059 | 2.96 | 58.99 | > 80 | 1.6 ± 0.2 | 1.1 ± 0.3 | 0.58 | 0.028 |
| MMV016838 | 4.83 | 54.88 | > 80 | 2.5 ± 0.2 | 1.2 ± 0.3 |  | 0.14 |
| MMV020623 | 3.27 | 78.49 | > 80 | 1.7 ± 0.2 | 1.3 ± 0.5 | 0.608 |  |
| MMV020710 | 2.53 | 97.53 | 72.2 | 3.3 ± 0.3 | 1.4 ± 0.5 | 0.79 |  |
| MMV637953 | 5.24 | 522.5 | > 80 | 2.6 ± 0.4 | 1.1 ± 0.0 |  |  |
| MMV676186 | 4.51 | 117.2 | > 80 | 1.8 ± 0.3 | 1.1 ± 0.3 |  |  |
| MMV676431 | 3.68 | 52.15 | 72.8 | 2.2 ± 0.2 | 1.1 ± 0.4 | 0.22 |  |
| MMV676597 | 4.00 | 93.34 | > 80 | 2.5 ± 0.3 | 1.0 ± 0.3 |  |  |
| MMV687146 | 4.99 | 49.74 | > 80 | 1.8 ± 0.2 | 1.1 ± 0.4 | 0.085 | 0.0019 |
| MMV688330 | 3.17 | 64.61 | > 80 | 1.5 ± 0.3 | 1.6 ± 0.6 | 0.56 | 0.055 |
| MMV688990 | 4.87 | 68.4 | > 80 | 2.3 ± 0.6 | 1.0 ± 0.1 |  |  |

*fu*_mic_ (fraction unbound) denotes the fraction of drug that was not bound to human microsomal protein at 0.25 mg/ml protein and 0.5 µM drug, pH 7.4, from (MMV, 2017). *fu*_mouse_ (fraction unbound) denotes the drug fraction that was not bound to mouse plasma protein, as evaluated by equilibrium dialysis at 37°C and 1 µM drug, from (MMV, 2017). *NormRate* was measured in the presence of drug alone; *NormRate*_BSA_ was measured in the presence of 60 µM BSA (Mean ± range/2, *n* = 2).
