# Supplementary material for: Screening for bilayer-active and likely cytotoxic molecules reveals bilayer-mediated regulation of cell function
Source: J Gen Physiol. 2023 Feb 10;155(4):e202213247. doi: 10.1085/jgp.202213247 (PMC9948646; doi:10.1085/jgp.202213247)
Supplement: Table S3 — shows on the average values for ALogP and PSA for drugs with low, intermediate, or high bilayer-modifying potency or cytotoxicity [file JGP_202213247_TableS3.docx]

**Table S3**: Average values for ALogP and PSA for drugs with low, intermediate, or high bilayer–modifying potency or cytotoxicity

| Bilayer–modifying potency | | | |
| --- | --- | --- | --- |
|  | | ALogP | PSA |
| *Low* | *NormRate* < 1.25 | 2.9 ± 1.6 | 78 ± 42 |
| ↕︎ | 1.25 ≤ *NormRate* < 1.50 | 3.6 ± 1.2 | 78 ± 33 |
| *High* | 1.50 ≤ *NormRate* | 4.1 ± 1.1 | 79 ± 49 |
| Likely cytotoxicity | | | |
|  | | ALogP | PSA |
| *Low* | 50 µM ≤ *CC*_20_ | 2.8 ± 1.9 | 83 ± 61 |
| ↕︎ | 10 µM ≤ *CC*_20_ < 50 µM | 3.6 ± 1.1 | 73 ± 36 |
| *High* | *CC*_20_ < 10 µM | 3.6 ± 1.4 | 81 ± 31 |
