# Supplementary material for: Screening for bilayer-active and likely cytotoxic molecules reveals bilayer-mediated regulation of cell function
Source: J Gen Physiol. 2023 Feb 10;155(4):e202213247. doi: 10.1085/jgp.202213247 (PMC9948646; doi:10.1085/jgp.202213247)
Supplement: Table S4 — shows on the odds for drugs being bilayer-modifying or cytotoxic vs. ALogP [file JGP_202213247_TableS4.docx]

**Table S4**: Odds for drugs being bilayer-modifying or cytotoxic vs. ALogP

| **A** | Number of drugs with | | Odds |  | **B** | Number of drugs with | | Odds |
| --- | --- | --- | --- | --- | --- | --- | --- | --- |
|  | *NormRate* < 1.25 | 1.25 ≤ *NormRate* |  |  |  | 50 µM ≤ *CC*_20_ | *CC*_20_ < 50 µM |  |
| ALogP ≤ 3 | 99 | 38 | 0.384 |  | ALogP ≤ 3 | 53 | 84 | 1.585 |
| ALogP > 3 | 108 | 157 | 1.454 |  | ALogP > 3 | 62 | 198 | 3.194 |

(**A**) The odds ratio for a drug being bilayer-modifying (having 1.25 ≤ *NormRate*) is 3.8-fold higher for drugs with ALogP > 3 than for drugs with ALogP ≤ 3; 95% CI: 2.4 – 5.9.

(**B**) The odds ratio for a drug being cytotoxic (having *CC*_20_ < 50 µM) is 2.0-fold higher for drugs with ALogP > 3 than for drugs with ALogP ≤ 3; 95% CI: 1.3, 3.1.
