# Supplementary material for: Screening for bilayer-active and likely cytotoxic molecules reveals bilayer-mediated regulation of cell function
Source: J Gen Physiol. 2023 Feb 10;155(4):e202213247. doi: 10.1085/jgp.202213247 (PMC9948646; doi:10.1085/jgp.202213247)
Supplement: Table S5 — shows the chemically similar drugs in the Pathogen Box [file JGP_202213247_TableS5.docx]

**Table S5**: Chemically similar drugs in the Pathogen Box

**Part A**: Pairs of drugs where the difference in *NormRate* is greater
than the sum of the uncertainties in the *NormRate*s

| Drug | *NormRate* | HepG2 *CC*_20_ (µM) | ALogP | PSA (Å^2^) |
| --- | --- | --- | --- | --- |
| 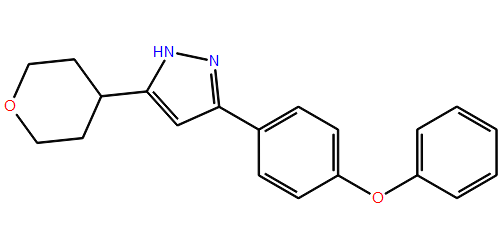  MMV687762 | 3.5 ± 0.6  1.4 ± 0.0 | 10.8  6.4 | 3.95  3.44 | 47.1  53.2 |
| 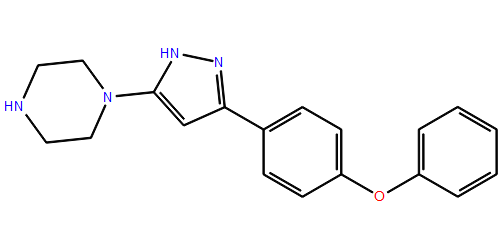  MMV687706 |  |  |  |  |
| 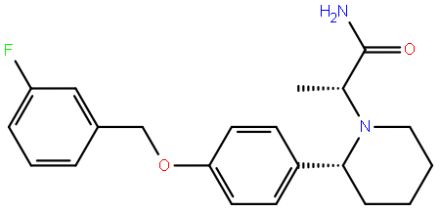  MMV676270  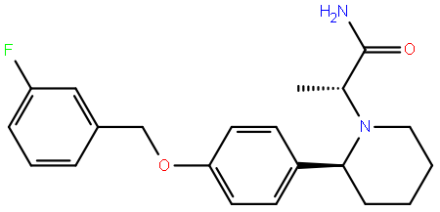  MMV676269 | 2.7 ± 0.4  1.2 ± 0.0 | 21.8  80 | 3.93  3.93 | 55. 6  55. 6 |
| 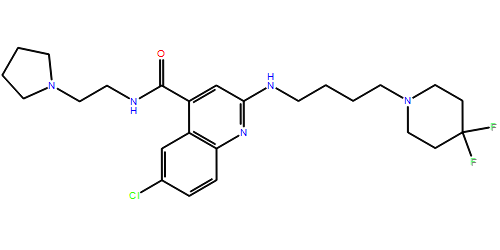  MMV667494  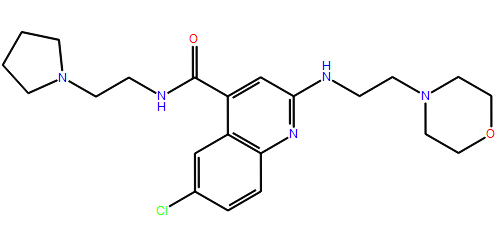  MMV634140 | 1.9 ± 0.4  1.2 ± 0.1 | 5.6  26.4 | 4.44  2.67 | 60.5  69.7 |
| 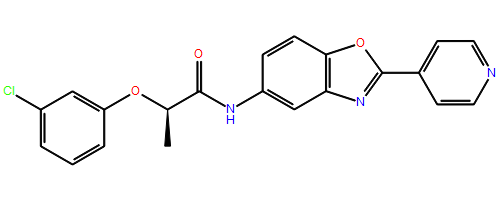  MMV688472  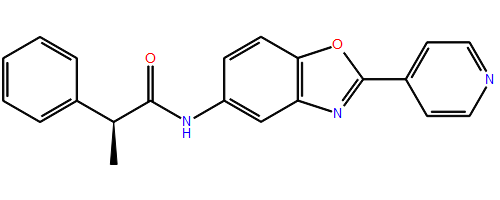  MMV688471 | 1.7 ± 0.2  1.1 ± 0.1 | 11  80 | 3.85  3.37 | 77.3  68.0 |

**Part B**: Pairs of drugs where the difference in *NormRate* is less
than or equal to the sum of the uncertainties in the *NormRate*s

| Drug | *NormRate* | HepG2 *CC*_20_ (µM) | ALogP | PSA (Å^2^) |
| --- | --- | --- | --- | --- |
| 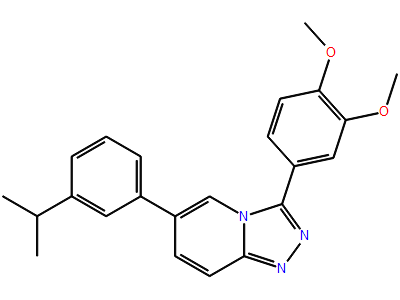  MMV688313  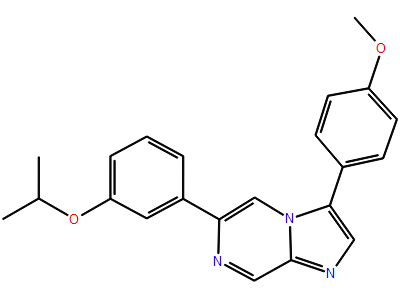  MMV688270 | 2.4 ± 0.1  2.0 ± 0.4 | 7.5  24 | 5.03  4.20 | 49.2  49.2 |
| 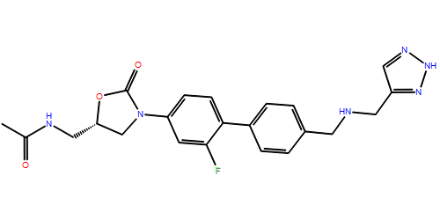  MMV688508  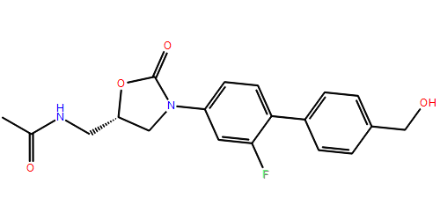  MMV688327 | 1.4 ± 0.3  1.2 ± 0.1 | 8.0  5.9 | 2.31  2.58 | 112.2  78.9 |
| 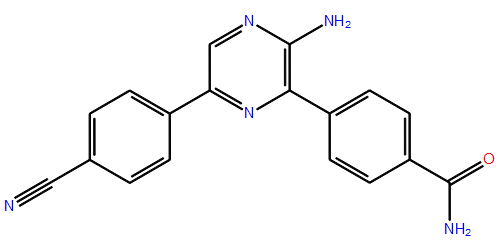  MMV085499  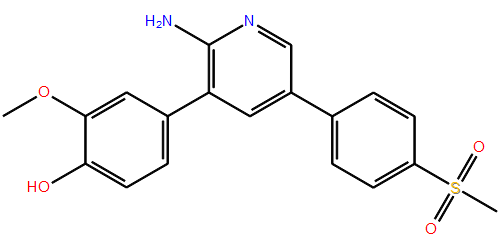  MMV010576 | 1.3 ± 0.1  1.1 ± 0.0 | 12  1.6 | 2.07  2.75 | 118  110 |
| 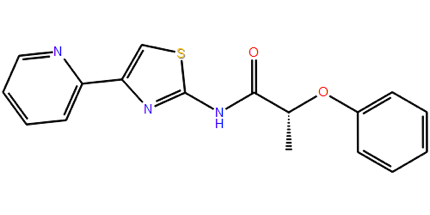  MMV676411 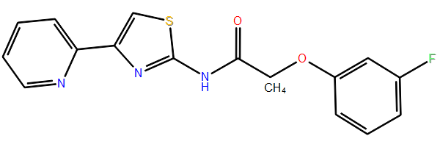 MMV676409 | 1.3 ± 0.2  1.2 ± 0.1 | 0.73  2.0 | 2.97  2.70 | 92.4  92.4 |
| 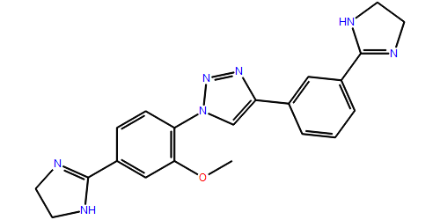  MMV688474  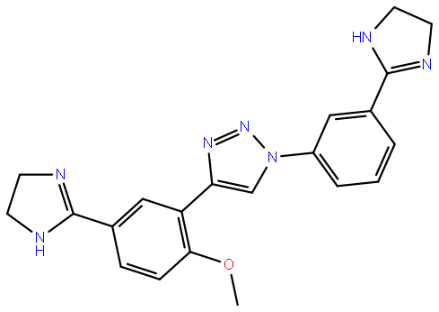  MMV688547 | 1.2 ± 0.0  1.2 ± 0.2 | 80  80 | 2.30  2.30 | 88.7  88.7 |
| 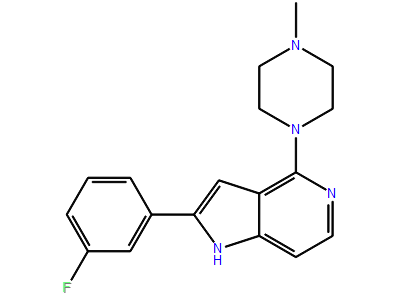  MMV393144  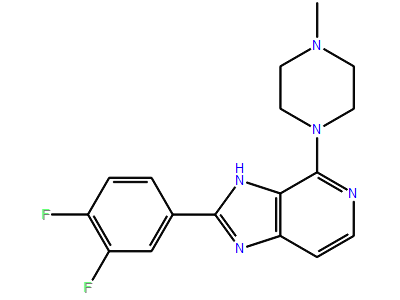  MMV676260 | 1.1 ± 0.0  1.1 ± 0.1 | 10.4  10.3 | 3.27  2.86 | 35.2  48.1 |
| 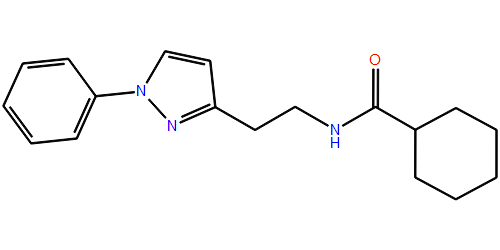  MMV688795  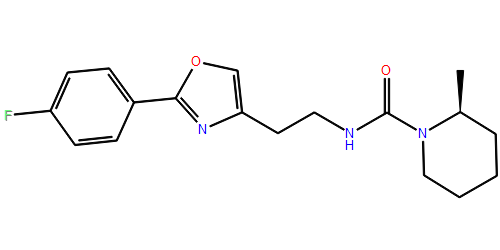  MMV688797 | 1.1 ± 0.0  1.1 ± 0.0 | 80  80 | 3.30  3.48 | 46.9  58.4 |
